# Opposing, spatially-determined epigenetic forces impose restrictions on stochastic olfactory receptor choice

**DOI:** 10.1101/2023.03.15.532726

**Published:** 2023-03-15

**Authors:** Elizaveta V. Bashkirova, Nell Klimpert, Ariel Pourmorady, Kevin Monahan, Christine E. Campbell, Jason M. Osinski, Longzhi Tan, Ira Schieren, Beka Stecky, Gilad Barnea, X. Sunney Xie, Ishmail Abdus-Saboor, Benjamin Shykind, Bianca Jones-Marlin, Richard M. Gronostajski, Alexander Fleischmann, Stavros Lomvardas

**Affiliations:** 1 Integrated Program in Cellular, Molecular and Biomedical Studies, Vagelos College of Physicians and Surgeons, Columbia University Irving Medical Center, Columbia University, New York, NY, 10032, USA; 2 Zuckerman Mind, Brain, and Behavior Institute, Columbia University, New York, NY, 10027, USA; 3 Department of Neuroscience, Division of Biology and Medicine and Robert J. and Nancy D. Carney Institute for Brain Science, Brown University, Providence, RI, USA; 4 Medical Scientist Training Program, Vagelos College of Physicians and Surgeons, Columbia University, New York, NY, 10032, USA; 5 Neurobiology and Behavior Graduate Program, Graduate School of Arts and Sciences, Columbia University, New York NY, 10027, USA; 6 Department of Biochemistry and Molecular Biology, Rutgers University, NJ, USA; 7 Department of Biochemistry, University at Buffalo and New York State Center of Excellence in Bioinformatics and Life Sciences, Buffalo, NY, USA; 8 Genetics, Genomics, and Bioinformatics Graduate Program, University at Buffalo and New York State Center of Excellence in Bioinformatics and Life Sciences, Buffalo, NY, USA; 9 Department of Bioengineering, Stanford University, CA, USA; 10 Beijing Innovation Center for Genomics, Peking University, Beijing, China; 11 Biomedical Pioneering Innovation Center, Peking University, Beijing, China; 12 Department of Biochemistry and Molecular Biophysics, Vagelos College of Physicians and Surgeons, Columbia University Irving Medical Center, Columbia University, New York, NY, 10032, USA

## Abstract

Olfactory receptor (OR) choice represents an example of genetically hardwired stochasticity, where every olfactory neuron expresses one out of ~2000 OR alleles in a probabilistic, yet stereotypic fashion. Here, we show that topographic restrictions in OR expression are established in neuronal progenitors by two opposing forces: polygenic transcription and genomic silencing, both of which are influenced by dorsoventral gradients of transcription factors NFIA, B, and X. Polygenic transcription defines spatially constrained OR repertoires, among which one OR allele may be selected for singular expression later in development. Heterochromatin assembly and genomic compartmentalization preferentially eliminate from this “privileged” repertoire ORs with more dorsal expression destinations, which are ectopically transcribed in neuronal progenitors throughout the olfactory epithelium. Our experiments identify early transcription as an “epigenetic” contributor to future developmental patterning and reveal how two spatially responsive probabilistic processes act in concert to establish deterministic, precise, and reproducible territories of stochastic gene expression.

## Introduction

The development of multicellular organisms relies on gene expression programs that are precisely regulated in space and time. To transform probabilistic biochemical reactions, such as transcription and translation, into reproducible differentiation processes, plants and animals convert individual cellular variability into predictable cell population averages. Yet, there are cases in biology where gene expression variability is desirable, as it generates diverse cellular identities that are difficult to obtain with deterministic gene regulation. For example, production of antibodies via VDJ recombination, and evasion of immunological responses by antigenic variation represent biological systems that seek utmost randomness[1, 2]. Other biological functions, however, benefit from balancing absolute determinism with complete randomness, producing biased stochasticity. Genetically encoded biased stochasticity is often observed in the nervous system, where gene expression choices generated by neurons must integrate into functional and reproducible circuits[3]. In fly ommatidia, for example, biased randomness preserves a ratio of photoreceptor neuron identities across animals[4], whereas in mammals, random Protocadherin promoter choice[5], was recently shown to obey spatial patterns in the mouse neocortex, assuring proper tiling between neighboring neurons[6].

Mammalian olfactory receptor (OR) gene choice provides an extreme case of hardwired biased randomness[7]. OR transcription starts in neuronal progenitors of the main olfactory epithelium (MOE), which transiently express 5–15 ORs out of >1,000 OR genes distributed across chromosomes[8–10]. As these progenitor cells differentiate into post-mitotic olfactory sensory neurons (OSNs), they switch from polygenic to monogenic and monoallelic OR transcription[11]. This transition is mediated by the assembly of a multi-chromosomal enhancer hub over a single OR allele[12–14], followed by the stabilizing effects of an OR-elicited feedback signal[15–19]. During this developmental progression, heterochromatic silencing[20] and genomic OR compartmentalization[21, 22] act together to assure that the non-chosen OR alleles will remain transcriptionally repressed for the life of the OSN. Interestingly, the position of the OSN across the dorsoventral (DV) axis of the MOE predisposes this singular transcriptional choice towards a group of 50–200 OR genes[23], providing reproducible topographic restrictions in OR expression. The anatomical segments of the MOE that express a specific collection of OR identities are known as “zones”, with their total number varying from 4 to 9, depending on the analyses and criteria used to define them[24–27]. Although zonal restrictions in OR expression have a well-established influence in the wiring of the olfactory circuit[28, 29], the mechanisms that bias this singular transcriptional choice towards specific OR identities remain unknown.

Here, we identified genetically encoded mechanisms that introduce topographic biases in OR gene regulation. We report that OSN progenitor cells from various MOE segments transcribe OR mixtures consisting of ORs with the corresponding or with more dorsal expression identities. Ectopic expression of dorsal ORs at the polygenic state of OR transcription is rectified during differentiation by preferential genomic silencing that is skewed towards ORs with more dorsal expression identities than the identity of the OSN. Patterns of polygenic OR transcription and genomic OR silencing are influenced by gradients of transcription factors Nfi A, B, and X [30]. Triple Nfi deletion reduces heterochromatic silencing and genomic compartmentalization from dorsomedial ORs and eliminates them from ventral ORs, whose transcription in progenitor cells also becomes extinguished. Furthermore, spatial transcriptomics revealed a dramatic expansion of dorsomedial OR expression towards the ventral MOE and reciprocal transcriptional reduction of ventral ORs in triple Nfi cKOs, suggesting that patterns of genomic OR silencing and polygenic OR transcription influence OR gene choice. Indeed, transcriptional induction of an OR allele in OSN progenitors biases the choice of this allele in mature OSNs (mOSNs) throughout the MOE. Strikingly, by modulating the levels of OR induction in progenitor cells we can restrict expression of an OR allele in more dorsal OSNs, where heterochromatic silencing and genomic compartmentalization is less prevalent. Thus, our studies reveal that position-responsive OR transcription in OSN progenitors acts as an “epigenetic” signal for future singular choice among the previously transcribed ORs. Moreover, our data suggest that polygenic transcription and heterochromatic silencing/genomic compartmentalization act as opposing regulatory “rheostats” that determine in a spatially influenced fashion the exact OR repertoire that is available for stochastic singular choice in mature OSNs.

## Results

### OSN progenitors co-transcribe an increasing number of zonal OR identities towards the ventral MOE

**Figure 1:**
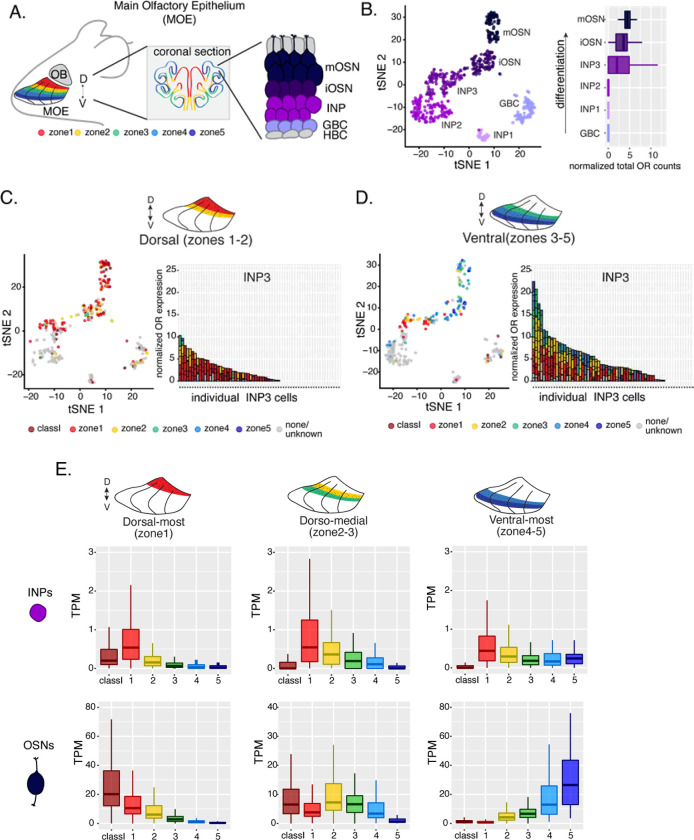
Polygenic transcription of OR genes in olfactory progenitors follows a zonal expression pattern. **(A)** Schematic illustrating OR zones along the dorsoventral axis, in whole mount views of the MOE (left) and coronal sections (middle). Zone1 (red) is the dorsal-most zone and zone5 (blue) is the ventral-most zone. Zoomed in view of a coronal sections (right) depicts MOE cell populations from different stages of OSN differentiation organized in a pseudostratified fashion from the basal (least differentiated) to apical (most differentiated) layers: HBC, horizontal basal cell; GBC, globose basal cell; INP, immediate neuronal precursor; iOSN, immature olfactory sensory neurons; mOSN, mature olfactory sensory neuron. **(B)** t-SNE clustering of single cells from FAC-sorted cell populations with Seurat based on the most variable genes showing the separation of single cells into 6 populations (left panel). The 6 populations were assigned cell identities using expression of known MOE markers[31] (See also Figure S1). Olfactory receptor expression is first detected in INP3 cells (right panel). **(C, D, left panel)** t-SNE clustering from FAC-sorted cell populations isolated from dorsal (zones 1–2 in C) or ventral (zones 3–5 in D) MOE microdissections. Cells are colored according to the zonal index of the most highly expressed OR. **(C, D, right panel)** Plots depicting zonal identities of all the ORs detected in individual INP3 cells from dorsal (C) or ventral (D) microdissections. Y axis shows OR expression in normalized UMI counts for different ORs (separated by black lines). On the X axis each point is a different INP3 cell. ORs are colored according to their zonal index. **(E)** OR expression by zonal index in olfactory progenitor INP cells (top) and mOSNs (bottom), determined with bulk RNA-seq, in cells isolated from dorsal-most (zone 1) (left), dorsomedial (zones 2–3) (middle), and ventral-most (zones 4–5) MOE microdissections (right). Note that INP and mOSN cells were FAC-Sorted from the same exact dissection, thus the mOSN OR expression patterns confirm the accuracy of the dissection.

The mouse MOE is divided into a limited series of stereotypic segments of OR expression that exhibit bilateral symmetry between the two nasal cavities (Figure 1A). In whole mount views, these segments present a dorsoventral (DV) segmentation pattern, with zone 1 being at the dorsal- and zone 5 at the ventral end of the MOE. Intricate invaginations of the MOE occurring during embryonic development and early postnatal growth, convolute this dorsoventral segmentation pattern, especially when viewing coronal sections of the MOE (Figure 1A). However, we will continue referring to the DV coordinates of each one of the five segments, or zones, as they correspond to their initial patterning during development.

Within each zone mOSNs express a single OR allele that is randomly chosen among 50–200 OR genes with proper zonal identities. However, before the onset of singular OR expression, mitotically active OSN progenitors, the immediate neuronal precursor (INP) cells, co-express, multiple lowly expressed ORs [8–10]. To determine whether zonal restrictions are operational from this polygenic stage of OR transcription, we performed deep scRNA-seq analyses of FAC-sorted OSNs and OSN progenitors from micro-dissected dorsal or ventral MOE segments (Supplemental Figure S1A). To enrich our plate-based scRNA-seq for cell populations of interest we used Mash1CreER; tdTomato; Ngn1-GFP triple transgenic mice (Supplemental Figure S1B). We injected P4 mice with tamoxifen, inducing permanent tdTomato expression, and then collected cells 48 hours later (Supplemental Figure S1B). From each dissection we isolated four major cellular populations corresponding to four successive differentiation stages, as previously described[31, 32]: GBCs (MOE stem cells), INPs (immediate neuronal precursors), iOSNs (immature OSNs), and mOSNs (Supplemental Figure S1C-D).

We first detect OR mRNAs in INP3 cells (Figure 1B), which consistently transcribe multiple ORs. Surprisingly, while dorsal INPs transcribe almost exclusively dorsal ORs, ventral INPs have complex OR mixtures consisted of every zonal identity (Figure 1C-D). With a 3 UMI cut off, we detect dorsal ORs in 43 ventral INP cells, and ventral ORs in only 29 of them, while dorsal INP cells express predominantly dorsal ORs (Figure 1C-D). Moreover, as the ventral INPs differentiate to iOSNs, dorsal OR transcription is replaced by ventral, zone-appropriate OR transcription, culminating to singular expression of an OR allele with the correct zonal identity (Figure 1D, Supplemental Figure S1). These observations were independently confirmed by bulk RNA-seq on FAC-sorted INP and mOSN cells extracted from trisected dorsal, medial, and ventral MOE. This bulk analysis showed that in every case INPs co-transcribe ORs with the correct and with more dorsal zonal identities, while further differentiation replaces dorsal ORs with ORs of the correct identity (Figure 1E). This immediately poses mechanistic questions about the process that shuts off dorsal ORs and enhances the transcription of the ORs expected to be expressed in each MOE segment.

### Heterochromatin eliminates ectopically expressed ORs along the dorsoventral MOE axis

**Figure 2:**
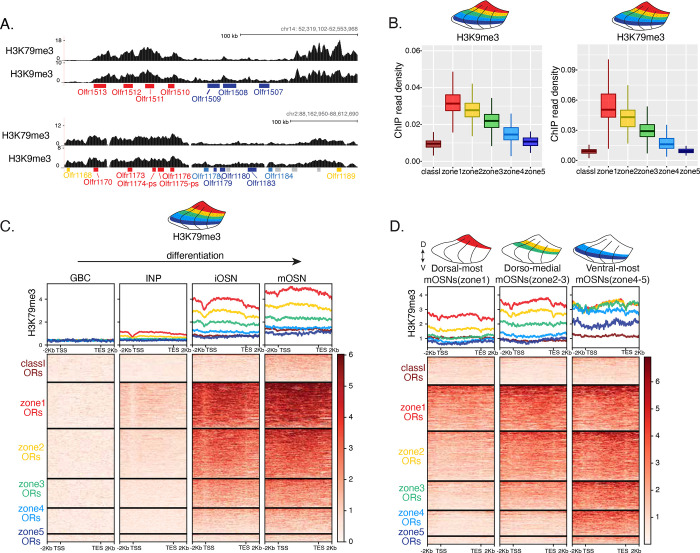
Heterochromatin deposition silences OR genes from lower zones. **(A)** Signal tracks of H3K9me3 and H3K79me3 native ChIP-seq from the whole MOE over two representative OR gene clusters that were selected because they harbor ORs from both zone 1 and zone 5. Below the signal track OR genes are colored according to their zonal index: zone1 ORs in red, zone2 ORs in yellow, zone 5 ORs in blue and ORs with unknown zonal index in gray. **(B)** H3K9me3 (left) and H3K79me3 (right) native ChIP-seq in the whole MOE. Box plots of read density over OR gene bodies separated by their zonal index depict a pattern of deposition that is high on dorsal-most expressed zone 1 OR genes, progressively decreases with more ventral zonal OR indexes, and is absent on class I ORs. **(C)** H3K79me3 native ChIP seq in GBC, INP, iOSN and mOSN populations shows onset of H3K79me3 deposition in INP cells. Each row of the heatmaps shows coverage over an OR gene body (separated into categories by their zonal index). (See also Supplementary Figure S2 for H3K9me3 heatmap). **(D)** H3K79me3 native ChIP-seq in mOSNs from zonally dissected MOE. Colored schematics above each heatmap depicts the zone of dissection. (See also Supplementary Figure S2 for H3K9me3 heatmap).

We previously showed that OSN differentiation coincides with heterochromatin-mediated OR gene silencing [20]. If heterochromatinization contributes to singular OR choice by eliminating every non-chosen OR transcribed in INPs, then in any MOE segment silencing should be preferentially applied to ORs expressed in that segment and to more dorsal ORs. Therefore, dorsal ORs (zone 1 ORs), which are expressed in INPs throughout the MOE, should have highest levels of heterochromatin, whereas ventral ORs (zone 5 ORs), transcribed only in ventral INPs, should have the lowest, with the rest of the OR repertoire having intermediate levels of heterochromatic marks. Visual inspection of ChIP-seq genomic tracks along OR gene clusters with mixed zonal constitution, reveals highest H3K9me3/H3K79me3 levels on the dorsal ORs and lowest on the ventral ORs of the cluster (Figure 2A). Aggregate ChIP-seq analysis of all the ORs grouped by zonal identities, corroborates the gradual reduction of H3K9me3 and H3K79me3 enrichment from dorsal to ventral ORs for the whole OR repertoire (Figure 2B). The only exception from this pattern is found at the dorsally expressed class I ORs, which rely on different regulatory mechanisms than the canonical class II ORs [33, 34] (Figure 2B). Finally, using the FACS-based strategy described earlier, we confirmed that both heterochromatic marks are predominantly deposited during the INP to iOSN transition, simultaneously with the transition from polygenic to singular, zonally appropriate OR expression (Figure 2C, Supplemental Figure 2A). Importantly, the descending pattern of heterochromatin enrichment from dorsal to ventral OR identities is preserved throughout differentiation.

We then asked if the patterns of heterochromatin deposition detected in mixed OSNs from the whole MOE are preserved in distinct zones. ChIP-seq in dorsal, dorsomedial, and ventral mOSNs showed that OR groups transcribed in INPs have higher heterochromatin levels than the ones that were not transcribed in that MOE segment (Figure 2D, Supplemental Figure S2B). Thus, most ORs are heterochromatic in ventral OSNs; dorsal and dorsomedial ORs are heterochromatic in dorsomedial OSNs; and only dorsal ORs have some heterochromatin in dorsal OSNs (Figure 2D). Although each zonal OR group is heterochromatic in the MOE segment that is expressed, it has lower enrichment of H3K9me3/H3K79me3 in its own segment than in more ventral segments, where it not chosen for stable expression. Thus, dorsal ORs have less heterochromatin in dorsal OSNs than the rest of the MOE, and dorsomedial ORs have less heterochromatin in dorsomedial OSNs than ventral OSNs. Similarly, at the ventral end of the DV axis, ventral ORs have less heterochromatin than dorsal and dorsomedial ORs. Detection of heterochromatin on ORs from the zonal group that is expressed is not counterintuitive, as only one OR allele from the ones co-transcribed will be eventually chosen, and the rest must be silenced (Supplemental Figure S2C). In other words, in every MOE segment, OR heterochromatinization is preserved only for the ORs that have the potential to be expressed and is not applied to more ventral ORs, which were not transcriptionally active in INPs. This is consistent with recent reports of heterochromatin marks being detected on trace amine-associated receptor (TAAR) genes only in TAAR-expressing OSNs and not the rest of the MOE (REF).

### DV gradient of OR gene compartmentalization follows patterns of heterochromatin assembly

**Figure 3:**
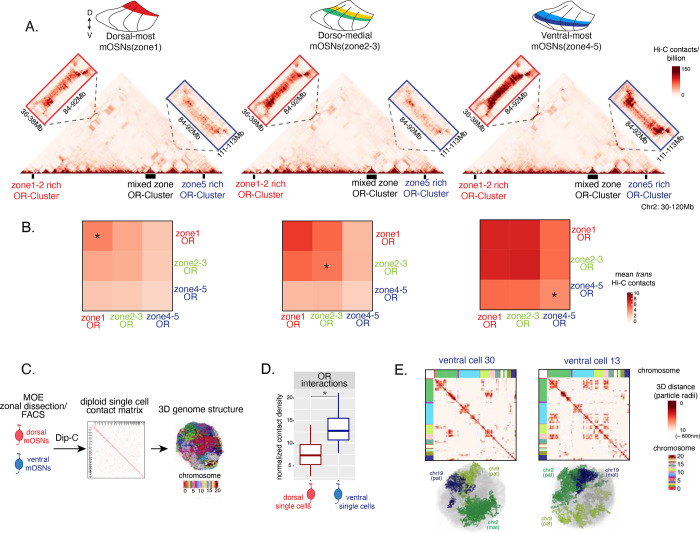
Zonal OR compartmentalization permits OR genes from more ventral zones to be recruited into the OR compartment. **(A)** *In situ* Hi-C contact matrices of a 90Mb region of chromosome 2 that contains 3 large OR gene clusters, depicted with orthogonal boxes under the contact matrices. HiC libraries were prepared from mOSNs FAC-sorted from dorsal-most (zone1), dorsomedial (zone2) and ventral-most (zone 4/5) MOE microdissections. For each contact matrix, we magnify the long-range cis contacts between the large OR cluster that contains ORs from every zone with the OR cluster that contains mostly zone 1–2 ORs (red box on the left) and the OR cluster that contains mostly zone 4–5 ORs (blue box on the right). All *cis* OR contacts increase from dorsal to ventral OSNs, but zone 4/5 ORs associate with the other ORs only in the most ventral OSNs (as noted by the HiC signal on the blue box). **(B)** Heatmaps of average interchromosomal Hi-C contacts between OR genes annotated by their zonal index at 50Kb resolution shows increased *trans* contacts in OSNs from more ventral zones. OR genes have a similar, intermediate frequency of contacts in the mOSN population where they are expressed, marked with an asterisk. **(C)** Dip-C on mOSNs from dorsal and ventral dissected MOE was used to generate haplotype resolved single cell contact matrices and 3D genome structures, as previously described[37]. **(D)** Analysis of Dip-C contact densities of interchromosomal contacts between ORs genes confirms that ventral mOSNs have increased OR compartment interactions (Wilcoxon rank sum p-value = 9.164e-11). **(E)** Single cell heatmaps of pairwise distances between OR genes generated from 3D genome structures in two ventral mOSNs show OR genes from different chromosomes intermingle in a different pattern in the two cells (top). For each cell, heatmaps are sorted by chromosome order and show all OR interactions within 10 particle radii (approximately ~600nm). Representative 3D structures showing the different positioning of three chromosomes (chr19, chr9 and chr2) in the two cells resulting in a different pattern of OR cluster contacts (bottom). See also supplemental Figure S3 for a complete map of all the Dip-C libraries.

Heterochromatic ORs converge into multi-chromosomal genomic clusters of extreme chromatin compaction that contributes to the effective and stable OR silencing [21]. We thus asked if the spatially determined pattern of OR heterochromatinization at the linear genome coincides with similar patterns of 3D genomic compartmentalization. *In situ* Hi-C in FAC-sorted OSNs from MOE segments along the DV axis revealed a striking resemblance between deposition of heterochromatic marks and genomic compartmentalization. For example, inspection of the genomic interactions between 3 OR clusters in chromosomes 2, shows that a cluster of ventral ORs is recruited to OR compartments only in ventral OSNs, where they are heterochromatic (Figure 3A). In contrast, the other two clusters, which are either enriched for dorsal ORs, or have mixed constitution, make strong genomic contacts with each other in all three MOE segments (Figure 3A). To expand this analysis to every OR, we measured the frequency of interchromosomal genomic interactions between ORs with different DV identities, reaching the same conclusion: Interactions between dorsal ORs is the default in every OSN, whereas compartmentalization for the remaining of the OR repertoire increases along the DV axis (Figure 3B). Intriguingly, as with levels of heterochromatin, we detect the following recurrent pattern of OR compartmentalization: every OR has intermediate HiC contact frequencies with other ORs in their expression segment, lower HiC contact frequencies in more dorsal segments, and higher HiC contacts ventrally.

The “intermediate” levels of heterochromatin enrichment and HiC contacts observed on ORs within their expression zone may reflect a less compact, transcription-compatible state of heterochromatin, or less frequent silencing of these ORs compared to more dorsal ORs. To distinguish between the two scenarios, we explored OR silencing at the single cell level using Dip-C[35–37]. We performed Dip-C in 48 dorsal and 48 ventral OSNs (Figure 3D). We used haplotype resolved data to compute distances of all genomic loci at 20kb resolution and generated 3D models for all cells (Figure 3C), as previously described[37]. Analyzing contact densities between OR loci, as well as distances in the 3D model we confirmed that OR compartments from ventral OSNs are larger and contain more ORs from more chromosomes than in dorsal OSNs (Figure 3D), consistent with our bulk HiC data. Importantly, “stronger” HiC contacts among dorsal ORs observed in bulk, represents increased number of dorsal ORs participating in OR compartments in each OSN, rather a closer distance between dorsal ORs within a compartment (data not shown). Thus, extrapolating Dip-C results to H3K9me3/H3K79me3 enrichment, we conclude that “intermediate” silencing levels of each OR group in their own zone likely reflects less frequent silencing than ORs from a more dorsal zone. In this note, OR compartmentalization is highly probabilistic, with each one of the 48 dorsal and ventral OSNs having unique maps of OR-OR genomic interactions (Figure 3E) (Supplemental Figure 3A, B). Thus, we propose that the balance between two probabilistic, yet DV-responsive processes, early transcription and genomic silencing defines the OR ensemble that is available for singular choice along the DV axis. To test this model, we sought to identify factors responsible for generating these remarkable patterns.

### NFI paralogues generate DV patterns in OR expression

**Figure 4:**
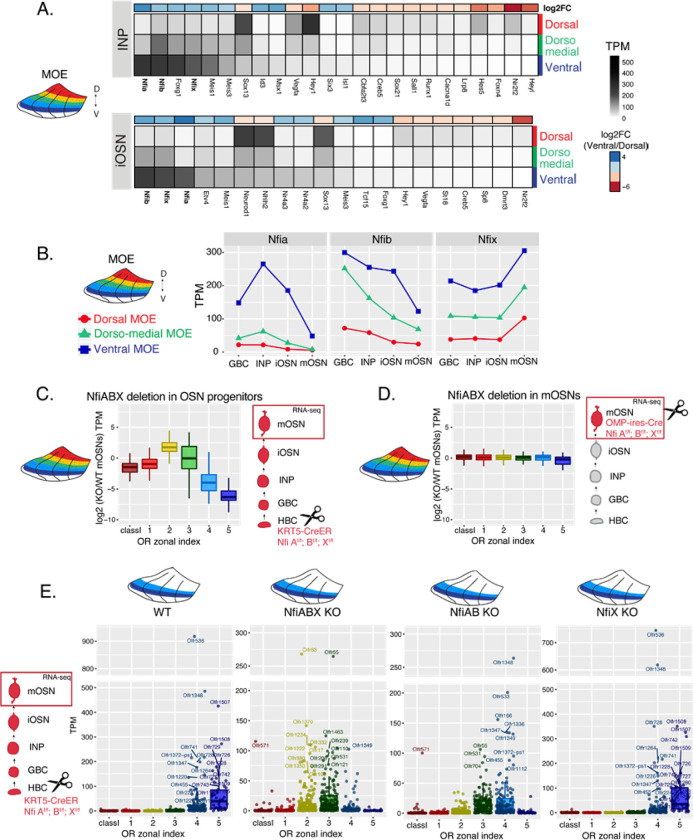
NFI paralogue gradients regulate zonal OR expression (A) Heatmaps showing the expression levels of differentially expressed transcription factors between dorsal, dorsomedial, and ventral cells during the INP and iOSN developmental stages. The shown transcription factors are significantly differentially expressed between dorsal and ventral cells with an adjusted p-value of <0.05, at least a three-fold change in expression, and an expression of at least 15 TPM. A broader list of transcription zonal factors is included in Table S1. The heatmaps is sorted based on expression in ventral cells and a color bar above each heatmap shows the log2 fold change in ventral cells relative to dorsal cells. (B) Expression levels of NFIA, NFIB, and NFIX at four stages of OSN development in dorsal cells (red), dorsomedial cells (green) and ventral cells (blue). (C, D) Comparison of OR gene expression in NFI ABX triple knockout and control cells from the whole MOE. NFI transcription factors are deleted either in progenitors (C) using a KRT5-CreER driver or in mOSNs (D) using an Omp-IRES-Cre driver (as described in Figure S4). At the schematic depiction of the experimental strategy at the right of each panel, scissors indicate the differentiation stage of the deletion, and red box the cell type that was FAC-sorted for RNA-seq analysis. (E) OR expression in NFI ABX triple knockout, Nfi AB double knockout, Nfi X knockout and control mOSNs from ventrally dissected MOE. Knockout was induced in progenitors with KRT5-CreER. Plots show a different pattern of OR gene transcription in the different genotypes. Quantification of differentially expressed ORs for each genotype shown in Figure S4.

We searched for transcription factors that have strong expression during the INP to iOSN transition that is graded across the DV axis of the MOE. NFI paralogues NFIA, B, and X have strong, DV-influenced expression in INPs that is preserved in iOSNs (Figure 4A, B and Supplemental Table 1). Specifically, NFIA and NFIB are expressed higher in ventral INPs and iOSNs, and NFIX is higher in ventral mOSNs (Figure 4B).These three members of the nuclear factor I (NFI) family of transcription factors control a plethora of developmental and cell specification processes[30, 38], and were previously implicated in OSN differentiation[39, 40]. Thus, we decided to explore genetically their contribution in the establishment of dorsoventral patterns of OR expression.

To interrogate the potential role of NFI A, B and X in zonal OR expression we deleted all three genes simultaneously using the Krt5CreER driver, which is expressed in quiescent stem cells of the MOE (HBCs). We crossed Krt5CreER; tdTomato mice to NFIA, B, X fl/fl mice[41], and induced recombination with tamoxifen. To force the quiescent HBCs to differentiate into OSNs, we ablated the MOE with methimazole and allowed 40 days for a complete restoration by the marked progeny of the NFI KO or control HBCs (Supplemental Figure S4A), as previously described[14]. RNA-seq analysis of the FAC-sorted OSNs from the whole MOE, revealed significant transcriptional reduction of ventral OR identities and reciprocal increase of dorsomedial ORs, with the transcription of the dorsal-most ORs being unaffected (Figure 4C). In contrast, triple NFI deletion only in mOSNs, with OMPiresCre has no measurable effects in OR expression (Figure 4D). To ask whether the reduced transcription of ventral ORs reflects a developmental defect of ventral OSN differentiation, versus a *bona fide* dorsalization of ventral OSNs, we performed RNA-seq in OSNs isolated specifically from ventral MOE microdissections. This experiment revealed ectopic expression of dorsomedial OR identities in place of the proper ventral ORs (Figure 4E), a result confirmed by immunofluorescence (IF) experiments (Supplemental Figure S4B,C). This transcriptional transformation of ventral OSNs satisfies the original criteria of homeosis [42], since the overall mOSN identity is not altered by the triple NFI deletion: Only 13 out of ~200 OSN-specific genes are significantly different between control and KO OSNs, and 117/207 non OR zone 4/5 enriched genes are still expressed in the ventral-most zones, acting as independent fiducial markers for our zonal dissection (Supplemental Figure S4E). The severity of this dorsomedial transformation depends on the number of NFI genes deleted, with the triple cKO expressing predominantly zone 2 and 3 ORs, double NFIA, B cKO expressing zone 3 and 4 ORs, and single NFIX cKO having almost wild type expression patterns of zone 4 and 5 ORs (Figure 4E, Supplemental Figure S4D).

### Spatial transcriptomics reveal widespread homogenization and dorsalization of the MOE upon triple Nfi deletion

**Figure 5:**
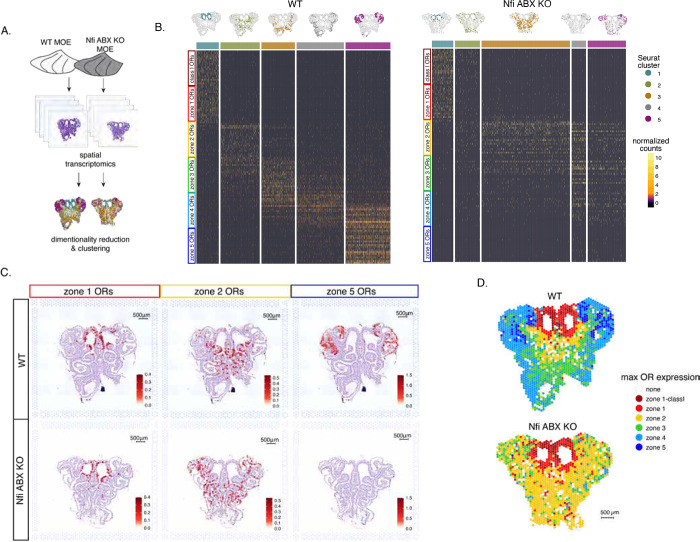
Spatial transcriptomics shows dorsalization and homogenization of the MOE upon Nfi A, B, and X deletion. **(A)** Schematic depicting our analysis pipeline: Spatial transcriptomics was performed on sections of control (wt) and Nfi ABX cKO MOE. Dimensionality reduction was performed, and spatial spots were clustered based on normalized expression of OR genes. **(B)** Heatmaps showing scaled, normalized expression levels of the top 20 highest expressed OR genes per zone in the control (wt) dataset. Unbiased neighborhood analysis and clustering grouped spatial spots into 5 clusters for both control (wt) and cKO MOE (depicted in distinct colors on the top of the heatmaps). Clustering of spatial spots in control (wt) samples reproduces anatomical zones, as spots within each cluster express OR genes with the corresponding zone index (left heatmap). We generated the same clusters in Nfi cKO samples (right heatmap). Although cluster 1 expresses exclusively zone 1 ORs, like in control MOEs, clusters 2–5 exhibit homogenous OR expression, with ventral expansion of zone 2/3 ORs, and reduced representation of zone 4/5 ORs. **(C)** Average normalized per-spot expression of the 20 highest expressed OR genes from zone1, zone2, and zone5 is overlaid against H&E histological image of control (wt, top) and Nfi cKO (bottom) MOE sections. Expression of zone1 OR genes is confined to the same anatomical region for both control and Nfi cKO sections. Zone2 OR gene expression is spread to more ventral regions in the Nfi cKO compared to control sections. Expression of zone5 OR genes is detected in fewer spatial spots and at a lower expression level in the knockout sample. **(D)** Spatial spots were assigned to a zone based on the highest summed normalized expression of OR genes. Zonal spot assignment of the control (wt) sample visually reproduces known anatomical zones. In the Nfi cKO sample, spots in the dorsal region had the highest expression of Class I and zone1 OR genes, similar to control sample. However, at the rest of the Nfi cKO MOE, most spots have a zone 2 OR identity. Spots assigned ‘None’ did not contain any OR transcripts and were excluded from cluster analysis.

To obtain a complete and unbiased understanding of the consequences of triple Nfi deletion in patterns of OR expression we deployed a spatial transcriptomic approach. Since our goal was to decipher zonal patterns of OR expression across the dorsoventral MOE axis without requirements for single cell resolution, we opted for the Visium Spatial Gene Expression workflow (10X Genomics)[43]. This workflow is ideal for interrogation of spatial OR expression in mOSNs, as OR mRNAs are highly abundant and readily detectable in most spatial spots that contain OSN mRNAs. For increased stringency, we only included spatial spots that include more than 2 OR genes and 3 OR transcripts. We analyzed 4 MOE sections from NfiA,B,X triple cKO mice and littermate controls, from two mice each (Figure 5A). Expression data on OR genes were normalized and integrated across replicates (see STAR methods). We performed PCA analysis, by which spatial spots were arranged in 5 clusters in control and cKO MOEs (Figure 5B). Interestingly, while dimensionality reduction and unbiased clustering generated OR clusters that correspond to zonal patterns of OR expression, i.e., each cluster contains ORs from a specific zone in control MOEs, only zone 1/class I ORs followed this correlation in cKO MOEs (Figure 5B). The other 4 clusters homogenously express zone 2–4 ORs, with expanded expression of zone 2 ORs in every cluster and loss of zone 5 OR detection. Thus, conditional triple Nfi deletion causes loss of spatial patterning for zone 2–4 OR genes and loss of expression for zone 5 ORs, without influencing the expression of zone 1 ORs.

To depict the effects of triple Nfi deletion in spatial patterns of OR expression we plotted the average OR expression per spatial spot of the top 20 most highly expressed OR genes for zones 1, 2, and 5. We then overlaid the corresponding values against the histological images of the control (wt) and Nfi ABX cKO MOEs (Figure 5C). As observed in the clustering and heatmap analysis, zone1 OR expression is confined to the same anatomical region for both samples. However, zone 2 OR expression in the cKO MOE extends beyond its defined anatomical region of the control MOE, and spreads to the ventral-most zones (Figure 5C). This expansion is also observed in the expression of individual zone 2 genes (Supplemental Figure S5A). In contrast, the top 20 zone 5 OR genes, while highly expressed in control MOEs, are almost undetectable in Nfi cKO MOEs (Figure 5C), consistent with our RNA-seq analysis. Olfr1507, the most highly expressed zone 5 OR, is undetectable in the cKO spatial spots (Supplemental Figure S5B), in agreement with our IF data. Finally, to obtain a more general understanding of the spatial transformations in OR expression patterning upon triple Nfi deletion, we assigned a zonal index of each spatial spot using the maximum normalized expression of all the OR genes detected in a spot (see STAR methods). Unlike control MOEs, where spot assignment reproduces zonal anatomical positions, most spatial spots in the cKO MOEs are assigned to zone 2, in a striking dorsalization and homogenization of the MOE (Figure 5D).

### NFI gradients control patterns of OR heterochromatinization and polygenic OR transcription

**Figure 6:**
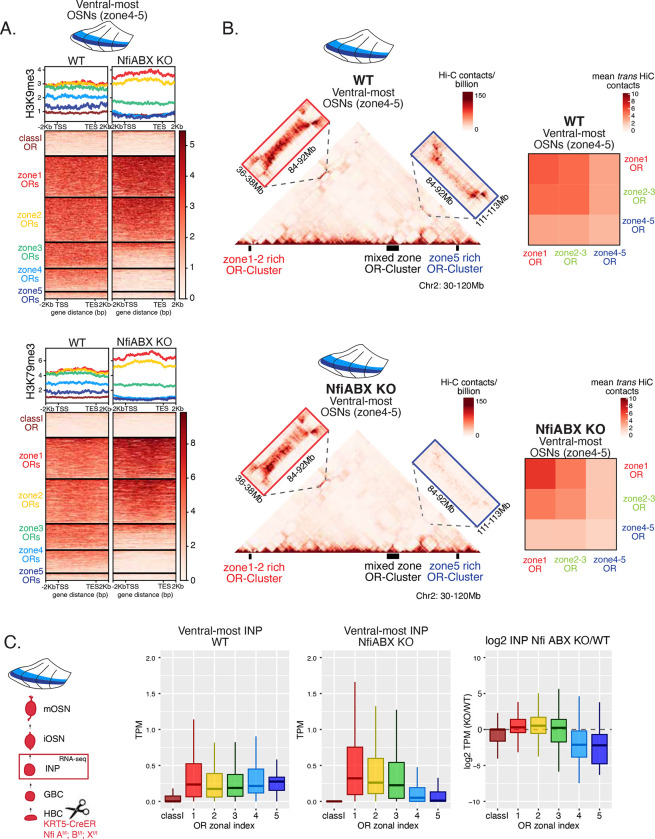
Nfi A, B and X regulate chromatin state and OR compartment formation (A) Native ChIP-seq for H3K9me3 (top) and H3K79me3 (bottom) in Nfi ABX knockout mOSNs from ventral MOE. Heatmaps of ChIP signal over OR genes show decrease of both histone marks on zone 3–5 index ORs in NfiABX knockout compared to control. Triple Nfi deletion was induced by KRT5-CreER (before OSN differentiation). (B) HiC in in Nfi ABX knockout mOSNs from ventral MOE. Left: In situ Hi-C contact matrices of a 90Mb region of chromosome 2 from control (top) and NFI ABX triple knockout (bottom) mOSNs, as described in Figure 3A. from ventral MOE show long-range cis interactions between 3 large OR gene clusters. Note that long range cis contacts the zone 4/5 enriched cluster and the mixed cluster dissipate in the triple Nfi cKO (bottom blue box), whereas the contacts of the mixed cluster with the zone 1/2 enriched cluster are preserved (bottom red box). Right: Heatmaps of average interchromosomal Hi-C contacts between OR genes annotated by their zonal index (as described in Figure 3B) in control (top) and triple Nfi cKO (bottom) mOSNs from ventral MOE. Trans contacts between zone 4/5 ORs dissipate, whereas trans contacts between zone 2/3ORs reach intermediate levels detected at “in zone” ORs. (C) OR expression by zonal index in INP cells isolated from ventral Nfi ABX knockout and control MOE. Nfi ABX INP cells were isolated as described in Figure S4. Log2FC of OR expression in Nfi ABX vs control INP cells, shows a decrease in expression of zone4–5 ORs (right).

We searched for a mechanistic explanation for the homeotic transformation of ventral OSNs in NFI cKO mice. Our experiments so far have identified 3 spatially responsive processes that may contribute to the dorsoventral patterning of OR gene choice: polygenic OR transcription in INPs, OR heterochromatinization and genomic compartmentalization during INP to iOSN transition. Thus, we explored the effects of triple NFI deletion in all three processes. First, we investigated the effects of NFI deletion in OR heterochromatinization by ChIP-seq and HiC on triple NFI cKO OSNs from the ventral-most MOE segments. ChIP-seq revealed an almost complete loss of heterochromatin from ventral ORs and small reduction in dorsomedial ORs in NFI cKO ventral OSNs (Figure 6A, Supplemental Figure S7E, F). Similarly, *in situ* Hi-C in control and triple Nfi KO OSNs from ventral MOE segments, revealed a strong reduction in the long-range *cis* and *trans* genomic contacts made by ventral ORs, and intermediate reduction for dorsomedial ORs (Figure 6B). Dorsal ORs, which are not affected transcriptionally, did not exhibit any change in ChIP-seq and HIC contacts. (Figure 6A, B). Strikingly, in both processes, heterochromatin assembly and genomic compartmentalization, the patterns observed in ventral OSNs upon Nfi deletion, are like the ones observed in dorsomedial OSNs from the control MOEs (Supplemental Figure S6A, B).

Finally, we explored the effects of triple Nfi deletion to the polygenic transcription of ORs in INP cells. We used a FACS-based strategy to isolate INPs from the ventral MOE followed by bulk RNA-seq as described earlier. Again, as with the results from ChIP-seq and HiC experiments, we detect a conversion toward the signatures observed in dorsomedial INPs, i.e., detection of only dorsal and medial ORs and depletion of ventral OR identities from the INP transcriptome (Figure 6C). Thus, our data reveal an unexpected correlation between OR transcription in INP cells, and two diametrically opposing gene expression outcomes in OSNs: silencing for the majority of the co-transcribed OR alleles and singular choice for one of them. Although transcription mediated silencing is widely deployed for the stable repression of repetitive elements, transposons and retroviruses that integrate in the genomes of various organisms, a transcription-mediated gene choice mechanism that transcends differentiation stages is uncommon. Thus, we devised a genetic strategy that would test the hypothesis that polygenic OR transcription is a pre-requisite for singular OR choice.

### Early OR transcription promotes OR gene choice in mOSNs

**Figure 7:**
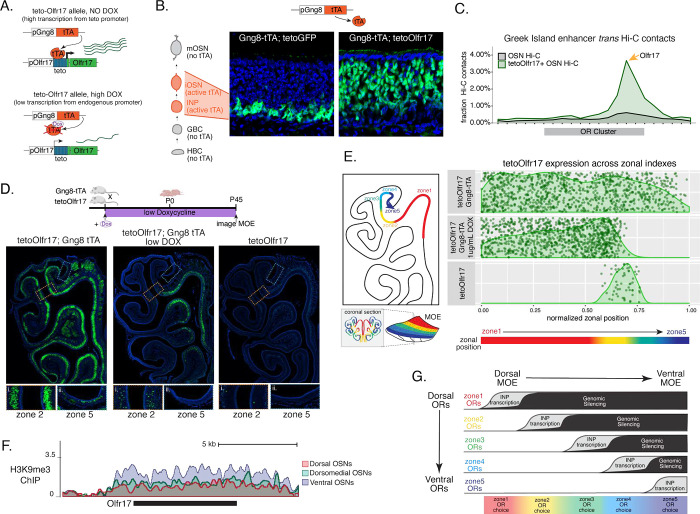
Genetic induction of OR transcription in olfactory progenitors determines OR choice in mOSNs **(A)** Genetic strategy for transcriptional induction of OR Olfr17 (a zone2 index OR) from its endogenous genomic locus. A genetically modified “tetoOlfr17” allele contains a tetO promoter immediately downstream of the endogenous Olfr17 promoter and an IRES GFP reporter after the coding sequence[44]. In the presence of tTA a high level of tetoOlfr17 is induced from the tetO promoter (top), while in the presence of a high amount of doxycycline tTA is inhibited and transcription is regulated by the endogenous promoter. See also Supplemental Figure S7A for information of the genomic locus of this Olfr17 allele. **(B)** tTA driven by the Gng8 promoter is expressed in INP and iOSN cells in the MOE[52]. When Gng8-tTA drives expression of tetoGFP transcription is driven only in progenitor cells located on the basal side of the MOE, where the tTA is expressed (left)[46]. In contrast, when Gng8-tTA drives expression of tetoOlfr17, expression persists in mature OSNs where tTA is no longer present (right). See also Supplemental Figure S7A,B for the sustained and widespread expression of this OR allele after 30 days in DOX treatment and for scRNA-seq data on Gng8 expression during OSN differentiation. **(C)** In situ Hi-C in tetoOlfr17 expressing cells shows enriched contacts with interchromosomal Greek Island enhancers over the Olfr17 locus, suggesting tetoOlfr17+ mOSNs are using endogenous mechanisms to sustain Olfr17 expression. **(D)** tetoOlfr17 expression in coronal sections of the MOE determined by GFP fluorescence. In the absence of tTA induction tetoOlfr17 expression occurs only in zone2 of the MOE (right); with high tTA induction in progenitor cells expression occurs throughout all zones of the MOE (left); and with low tTA induction in progenitors, due to the addition a low amount of doxycycline, expression occurs in zone2 and spreads dorsally to zone1 (middle). Zoom in shows tetoOlfr17 expression in its native zone2 (i) and ectopic expression in the most ventral zone 5 (ii). Mice on low doxycycline treatment were provided doxycycline at 1ug/ml in water throughout gestation and postnatal life. See Supplemental Figure S7C for the Dox administration protocol. **(E)** Quantification of tetoOlfr17 expression relative to a normalized zonal position (illustrated on the left) in tetoOlfr17 (bottom), tetoOlfr17 with Gng8tTA driver (top), and tetoOlfr17 with Gng8-tTA driver on low doxycycline (middle). 6 sections from two replicates were analyzed for tetoOlfr17 with Gng8-tTA; 9 sections from two replicates were analyzed from tetoOlfr17 with Gng8-tTA and low doxycycline; 29 sections from two replicates were analyzed for tetoOlfr17 without tTA. The plot displays a maximum of 1000 cells randomly selected for each condition. **(F)** H3K9me3 native ChIP signal over the Olfr17 locus in mOSNs from dorsal MOE (red), dorsomedial MOE (green) and ventral MOE (blue) shows a higher level of heterochromatin in ventral MOE. **(G)** Model of OR choice in each zone of the MOE, regulated by the interplay of low level polygenic transcription in INP cells which defines the OR repertoire that can be chosen in each zone, and heterochromatic silencing, which prevents ectopic expression of more dorsal ORs. See also Supplemental Figure S7D, E for ChIP-seq analyses on zone 4/5 INP cells from control and Nfi cKO INP cells.

We manipulated OR transcription using a genetically modified Olfr17 allele with a tetO promoter inserted immediately downstream of its transcription start site[44]. This allele enables strong transcriptional activation of Olfr17 from the endogenous genomic locus under the control of tTA (Figure 7A, Supplemental Figure S7A). Olfr17 expression is monitored by an iresGFP reporter inserted immediately downstream of the Olfr17 translational stop codon (Figure 7A). To induce transcription of this tetO-Olfr17iGFP OR allele in INPs and iOSNs, we used Gng8-tTA transgenic mice. Gng8 is expressed predominantly in INPs, downregulated in iOSNs, and completely shut off in mOSNs. Consistent with the expression properties of Gng8 and previous reports[45, 46], we only detect GFP in the basal MOE layers of Gng8-tTA; tetO-GFP mice (Figure 7B), which are enriched for INPs and iOSNs. However, when we cross the same Gng8-tTA driver to tetO-Olfr17iGFP mice, we detect widespread GFP signal in apical MOE layers, which contain predominantly mOSNs (Figure 7B). Since there is no tTA expression in mOSNs, we reasoned that the INP/iOSN-induced tetO-Olfr17iGFP allele is chosen for expression by the endogenous transcriptional machinery responsible for singular OR choice. Indeed, HiC experiments of these OSNs revealed that Greek Islands, the intergenic OR enhancers that converge over the chosen OR allele[12–14], are recruited specifically to the tetO-Olfr17iresGFP allele (Figure 7C), explaining the sustained expression of this OR in mOSNs. Furthermore, high doxycycline diet to these mice for 30 days fails to extinguish tetO-Olfr17iresGFP expression in mOSNs (Supplemental Figure 7), in support of the notion that transcriptional induction of Olfr17 in INPs/iOSNs, signals for the preferential choice of this OR in mOSNs. In contrast, ChIP-seq experiments on GFP-negative OSNs, which do not express this OR allele, did not reveal a significant increase of heterochromatin on the P2 locus (data not shown).

Intriguingly, transient induction of Olfr17 transcription promotes preferential choice of this OR throughout the MOE, rather than only in zone 2, were Olfr17 is normally expressed (Figure 7D). In fact, the vast majority of mOSNs from zones 1 to 4 are GFP^+^, and only in the ventral-most zone 5 we detect a more sporadic pattern of ectopic Olfr17 choice (Figure 7D, E). We hypothesized that reduced frequency of ectopic Olfr17 expression in the most ventral segment reflects the fact that heterochromatin levels and genomic compartmentalization of this dorsomedial OR allele is highest at this MOE segment even at the INP stage (Supplemental Figure S7E). This immediately suggests that the balance between transcriptional activation heterochromatic silencing during INP to iOSN transition determines whether an OR can be chosen for singular expression. If this hypothesis is correct, then reducing Olfr17 transcription in INP/iOSN cells, should preferentially prohibit ectopic Olfr17 expression in ventral MOE segments, where heterochromatic silencing is stronger. To test this, we pharmacologically manipulated tTA activity using low levels of doxycycline (1mg/Kg) administration throughout the life of the mouse (Supplemental Figure 7A), which reduce but do not eliminate tTA-driven transcription. Remarkably, mice that were subjected to this doxycycline regimen, continue to frequently express Olfr17 in dorsal mOSNs (zones 1–2), but not in mOSNs from more ventral MOE segments (zones 3–5) (Figure 7D, E), where heterochromatin levels on this OR allele are highest (Figure 7F). Thus, we can manipulate the zonal expression of an OR allele in mOSNs, by pharmacologically modulating the frequency and levels of transcriptional activation in INP/iOSN cells.

## Discussion

We uncovered a mechanism by which a random transcriptional process becomes skewed towards specific outcomes, transforming the relative position of a neuron across the dorsoventral axis of the MOE into biased OR gene choice. The solution to the perplexing segmentation of the MOE into distinct and reproducible territories of OR expression is the following: polygenic OR transcription in neuronal progenitors highlights a small group of ORs that can be chosen for singular expression later in development (Figure 7G). In each MOE segment this OR mixture includes ORs that should be expressed in mOSNs of the segment, as well as ORs that are only expressed in more dorsal MOE segments (Figure 7G). As these progenitor cells differentiate into iOSNs, heterochromatic silencing preferentially decommissions from this mixture more dorsal ORs, and with lower efficiency ORs that could be expressed in the segment, biasing this singular choice towards a spatially appropriate OR repertoire (Figure 7G). Our scRNA-seq analysis revealed two vectors in the determination of the OR ensemble that is co-transcribed in each OSN progenitor: chance, as every OR combination is unique, and determinism, as the overall zonal identities of the co-transcribed OR mixtures are informed by the position of the progenitor cell. Similarly, Dip-C revealed that genomic silencing also follows skewed patterns, eliminating preferentially ORs with more dorsal expression signatures than ORs that could be expressed in each zone. The final product of these opposing probabilistic “rheostats” is the generation of gene expression programs that may not have sufficient resolution to determine which OR will be chosen in every OSN but are precise enough to generate reproducible expression territories for each one of the ~1400 OR genes.

We identified gradients of transcription factors Nfi A, B, and X as partial orchestrators of the dorsoventral patterning of OR expression, which they establish as follows: they contribute to the silencing of dorsomedial ORs (zone 2 and 3 ORs); they activate both polygenic transcription and silencing of ventral ORs (zone 4 and 5 ORs); and they have no influence on the expression of dorsal-most ORs (class I and Zone 1 ORs). Given that Nfi factors are predominantly known as regulators of embryonic and adult stem cell biology[41, 47], it is surprising that in the olfactory system their deletion does not interfere with the maintenance of stem cell populations, but with the OR expression patterns in post-mitotic, fully differentiated mOSNs. Interestingly, triple NFI deletion after the onset of singular OR choice, has no effect in OR patterning, consistent with the emerging model that OR specification takes place exclusively at the INP to iOSN transition, and the notion that these patterning factors are not required for maintenance of OR transcription. Thus, we speculate that singular OR gene choice in OSNs can be executed by the common nucleoprotein complex of Lhx2/Ebf/Ldb1 bound to the multi-enhancer hub, consistent with the fact that we detect hubs of similar constitution associating with active ORs in different zones[14].

A question emerging from these observations is why not use the same transcription factor gradients to regulate both polygenic and monogenic OR transcription? The answer is likely related to the absolute requirement for transcriptional singularity: transcription factor gradients can transcribe specific OR mixtures in a DV-responsive fashion, but they cannot activate only a single OR promoter among the many they can bind to. But even if singularity was achievable by transcription factor combinations and the OR-elicited feedback, OR promoters with the strongest binding motifs would be consistently chosen first, excluding ORs with weaker promoters in a “winner takes all” model. This would result in preferential choice of specific ORs, reduced diversity in OR representation, and a narrower sensory spectrum for the olfactory system. With the process revealed here, the most frequently activated OR promoter in INPs/iOSNs has only a 5–10% chance of being chosen for singular expression in OSNs. Thus, by segregating OR gene regulation into two stages, polygenic transcription in progenitor cells and singular choice in OSNs, the olfactory system can impose deterministic biases while assuring equitable receptor representation. Of course, this system has limitations in preserving transcriptional equity: artificial transcriptional induction of an OR allele in OSN progenitors under the powerful tetO promoter, bypasses these constraints and results in biased choice of this allele in most mOSNs. This immediately suggests that *cis* OR regulatory elements are subject to selective pressure that preserves their weak transcriptional activation properties, explaining why robust OR transcription in mOSNs requires assembly of interchromosomal multi-enhancer hubs.

In this note, zones may also have evolved to satisfy the requirement for distributed OR representation: if dorsal-most ORs, which are detected in every OSN progenitor regardless of DV origin, have the most frequently activated promoters, then silencing them in more ventral MOE segments assures that other OR identities will also have the chance to be expressed. Consistent with this model, is the observation that mutations on the Lhx2 or Ebf binding sites of the promoter of dorsal OR M71, result in less frequent and more ventral M71 expression patterns[48]. Thus, DV segmentation of the MOE may serve as a mechanism that prevents ORs with stronger differences in promoter strength from competing for singular expression, assuring that every OR is expressed at meaningful, for odor perception, frequencies. In addition, as our spatial transcriptomic data showed, zonal regulation assures that ORs are expressed in a reproducibly patterned fashion in the MOE. While in wild type mice unbiased machine learning approaches identify at least 5 distinct OR expression patterns, in the triple Nfi cKO mice these patterns become intermixed for all but zone 1 ORs. With recent observations arguing that individual mitral cells, the second order neurons in the olfactory circuit, have patterned projections in the brain[49], non-random OR expression in the MOE may contribute to putative hardwired components of odor perception and valance[50].

### Polygenic OR transcription as the arbiter between OR gene silencing and OR gene choice

A peculiar feature of the OR gene family that had emerged from our past work is that OR gene silencing is highest in the very cells that express ORs[20]. Our zonal analysis further strengthened this intriguing correlation, as both H3K9me3/H3K79me3 and genomic compartmentalization in each MOE segment are strongest on OR groups that are transcriptionally active during OSN differentiation. A fascinating implication from this observation is that early OR transcription is the signal for both genomic silencing and singular choice. Although the former is only implied by the strong correlation between OR transcription in OSN progenitors and genomic silencing, the latter is experimentally supported by the striking observation that strong transcriptional induction of Olfr17 at the INP/iOSN stage results in strong recruitment of the Greek Island hub, and stable choice of this OR allele in most mOSNs throughout the MOE. Such a mechanism of promoter choice influenced by spatially-determined early transcription, could also explain the recent demonstration that clustered Pcdh choice, which is regulated by antisense transcription[51], abides to spatial restrictions in the neocortex[6].

How could two fundamentally opposite gene expression outcomes be encoded on the same molecular feature? We propose that the timing and levels of transcriptional induction could be the arbiters between genomic silencing and singular choice. ORs that are transcribed first in the INP stage, when the Greek Island hub cannot yet form due to the continuous expression of Lamin b receptor[21], are most likely to be silenced. OR alleles activated during the assembly of the multi-enhancer hub, at the INP to iOSN transition, may compete for hub recruitment. The OR allele that will first associate with a multi-enhancer hub, will be stably protected from heterochromatic silencing, possibly due to the significantly increased rates of OR transcription, whereas the other co-transcribed ORs will succumb to heterochromatic silencing. If timing and rates of OR transcription determine whether an OR allele will be silenced or chosen, then an OR allele that is highly transcribed in both INP and iOSN stage should evade silencing and dominate the competition for hub recruitment, explaining the striking expression pattern of the tTA-induced Olfr17 allele. Thus, according to this model, in each OSN ORs with more dorsal identity will be transcribed first, because they have stronger promoters, and therefore will become silenced in higher frequency; ORs with the correct zonal identity will be transcribed later, with a chance to associate with the Greek Island hub, explaining why one is chosen and the rest are silenced; ORs with more ventral identities will not be transcribed at all, thus, will not be silenced but also will not be chosen. In other words, singular OR transcription may not depend on the silencing of every single OR in the genome: by encoding silencing and stable choice with the same exact molecular feature, OSNs choose one and silence a small fraction of the whole OR repertoire in each nucleus- the rest are not relevant.

### Limitations of this study

Our experiments did not clarify whether NFI proteins bind directly on OR promoters, or act indirectly by activating other transcription factors and chromatin modifying enzymes. Although there is a statistically significant enrichment of NFI motifs on zone 4/5 OR promoters compared to the other OR promoters (data not shown), we were not able to detect direct binding of NFI proteins on these promoters, which is expected since these promoters are active in less than 1% of the cells. Given that our studies provide the mechanism by which NFI gradients establish zonal boundaries, via polygenic OR transcription and chromatin-mediated silencing, answering this question is not essential for understanding the mechanism of dorsoventral patterning of OR expression. A second limitation of this study is that it did not reveal the mechanisms that regulate the expression of the dorsal-most ORs (Zone 1 ORs), as NFI deletion had not effects in the expression and chromatin regulation of these OR genes. However, having revealed the regulatory logic whereby these patterns are established, we expect that other transcription factors with zonal expression patterns identified here, regulate early transcription and silencing of these genes across the MOE.

## STAR Methods

### EXPERIMENTAL MODEL AND SUBJECT DETAILS

Mice were treated in compliance with the rules and regulations of IACUC under protocol number AC-AAAT2450 and AC-AABG6553. Mice were sacrificed using CO_2_ following cervical dislocation. A complete list of mouse genotypes used for every experiment is in the Table2. Mash1-CreER (also known as Ascl1^CreERT2^)[53]; Ngn1-GFP[20] and Cre inducible tdTomato reporter (also known as *B6N.129S6-Gt(ROSA)26Sor*^tm1(CAG-tdTomato*,EGFP*)Ees/J^ )[54] mice were used to isolate four cell types in the olfactory lineage (GBC: tdTomato+ GFP−, INP: tdTomato+ GFP+, iOSN: tdTomato− GFP+ (bright), and mOSN: tdTomato+ GFP dim) by sorting cells 48 hours after tamoxifen injection. GFP bright and dim cells from Ngn1-GFP pups (P6) were also used to isolate a mix of INP/iOSN cells and mOSN cells respectively. Omp-ires-GFP[18] mice were used to isolate mature OSNs from adult (> 8-week-old) mice. In order to obtain zonal iOSNs and mOSNs, Olfr1507-ires-Cre[18] and tdTomato alleles were crossed in with either Ngn1-GFP or Omp-ires-GFP alleles to aid in zonal dissection (by labeling Ollfr1507+ expressing cells in zone5).

Early knockout of Nfi A, B, and X (NfiABX) in horizontal basal cells (HBSs: the stem cell of the olfactory epithelium) was achieved by crossing NfiA fl/fl NfiB fl/fl and NfiX fl/fl triple conditional alleles, described in [41], with KRT5-CreER[55] and tdTomato. Adult mice (> 8-week-old) had deletion of NfiABX in horizontal basal cells induced with 3 intraperitoneal injections with tamoxifen (24 hours apart). Ten days after the first injection, the olfactory epithelium was ablated with one intraperitoneal injection of methimazole, inducing proliferation of the HBCs and regeneration of a Nfi ABX knockout olfactory epithelium. The olfactory epithelium was allowed to regenerate for 40 days before collecting the MOE and FAC-sorting the tdTomato+(dim) cell population, which contains a mixture of mostly mOSNs and some INP and iOSN cells, as described in detail in [14]. For some experiments Omp-ires-GFP was crossed in to ensure all cells collected were mOSNs. To collect knockout INP cells the olfactory epithelium was only allowed to regenerate for 8 days before collecting the MOE and FAC-sorting the tdTomato+(dim) cells. Late knockout of Nfi ABX in mOSNs was achieved by crossing NfiA, B, and X triple conditional alleles with tdTomato and Omp-ires-Cre, and FAC-sorting tdTomato+ cells from adult mice. Complete list of all the mouse genotypes can be found in Table 2.

Induction of Olfr17 was achieved by crossing tetoOlfr17 mice, described in (reference), with Gng8-tTA mice (reference). To assess stability of tetoOlfr17 expression after induction, adult mice >8 weeks were placed on a diet containing high doxycycline—200mg/kg (Bio Serv, S3888)—for 35 days. To achieve a lower level of tetoOlfr17 induction, tetoOlfr17 mice were crossed with Gng8-tTA mice while being kept on a low amount of doxycycline in water—1ug/ml (Sigma Aldrich, D9891)—as described in (insert reference). Mice were kept on doxycycline water throughout gestation and postnatal life, until collecting the MOE for analysis from mice > 6 weeks old.

## METHOD DETAILS

### Zonal Annotation:

OR genes were assigned a zonal annotation (referring to their native zone of expression) based on [23]. We generated bins from the continuous data by rounding to the nearest integer. There are a total of 1011 ORs with known zonal annotation. Of these, 115 are ClassI ORs, of which nearly all are expressed in zone1, and 896 are ClassII ORs, of which 261 are expressed in zone1, 283 in zone2, 164 in zone3, 144 in zone4 and 44 in zone5.

### Zonal dissection of the olfactory epithelium:

We used the fluorescent signal in Olfr545-delete-YFP[56] (zone 1 OR), Olfr17-ires-GFP [18](zone 2 OR), and Olfr1507-ires-GFP[18] (zone 5 OR) mice to practice dissections of dorsal (zones 1) MOE, dorsomedial (zone 2–3) MOE, and ventral (zone 4–5) MOE, respectively. Upon obtaining an accurate understanding of the zonal boundaries in the MOE we performed zonal dissections without the use of these fiduciary markers. Accuracy of dissections was confirmed by RNA-seq. For some experiments Olfr1507-ires-Cre and tdTomato reporter was crossed in to assist with accurate ventral MOE dissection (see Table 2.)

### Fluorescence-activated cell sorting

Cells were prepared for FAC-sorting as previously described in[14] by dissociating olfactory epithelium tissue with papain for 40 minutes at 37°C according to the Worthington Papain Dissociation System. Cells were washed 2x with cold PBS before passing through a 40-um strainer. Live (DAPI-negative) fluorescent cells were collected for RNA-seq and native ChIP-seq. Alternatively, for Hi-C cells were fixed for 10 minutes in 1% formaldehyde in PBS at room temperature, quenched with glycine, and washed with cold PBS before sorting fluorescent cells. Alternatively, for Dip-C, cells were fixed in 2% formaldehyde in PBS at room temperature for 10 minutes, inactivated with 1% BSA, and washed with cold 1% BSA in PBS before sorting fluorescent cells. All cells were sorted on a BD Aria II.

### Single cell RNA-seq in olfactory lineage cell types

Mash1-CreER; tdTomato; Ngn1-GFP pups (ages P2-P4) were injected with tamoxifen and olfactory epithelium was collected after 48 hours. The tissue was dissected into ventral (zone 3–5) and dorsal OE (zone1–2) sections, from which GBC (tdTomato+, GFP−), INP (tdTomato+, GFP+), iOSN (tdTomato−, GFP+ bright) and mOSN (tdTomato-, GFP dim) cells were sorted into 384 well plates (split between the cell types). Each well of the 384 well plate had unique cell and molecular barcodes. Library preparation and sequencing was performed in collaboration with the New York Genome Center (NYGC) using a TSO approach for library preparation and sequenced on HiSeq2500. Reads were aligned to the mm10 genome according to the Drop-seq[57] pipeline (http://mccarrolllab.org/dropseq/), which uses STAR for alignment, and discarding multi mapped reads with Samtools -q 255. Aligned single cells had a median of 133,686 unique transcripts (UMIs) and 2,331 genes per cell (detected with a threshold of at least 3UMI). Experiment was performed in biological replicate, resulting in 764 cells, from which we discarded cells with less than 1000 genes and 20,000 UMIs, resulting in 669 cells. We further filtered for cells that contained less than 5% mitochondrial reads, resulting in 591 cells used for analysis. We used Seurat v3 to normalize counts and cluster single cells, resulting in 5 populations. Clusters were assigned a cell-type based on expression of known olfactory lineage markers. We used the default setting of genes expressed in at least 3 cells for clustering but changed it to 1 when looking at OR expression (since expression of any OR out of > 1000 genes is a rare event). For all OR expression analysis we used a threshold of 3UMI for an OR to be considered expressed.

### Bulk RNAseq in olfactory lineage cell types

GBC, INP, iOSN and mOSN were isolated from Mash1-CreER; tdTomato; Ngn1-GFP pups as described above with the tissue being dissected into a ventral (mostly zone 4–5), dorsal OE (mostly zone1) and a central section (that is enriched for zone2–3). The experiment was performed in biological replicate. RNA was extracted from FAC-sorted cells using Trizol and libraries were prepared with Nugen NuQuant RNA-seq library system and sequenced 50PE on HiSeq2500 or 75PE NextSeq (and trimmed to 50bp before aligning). Cutadapt was used to remove adapter sequences and reads were aligned to the mm10 genome with STAR. Samtools was used to select high mapping quality reads (-q 30). Normalization, calculation of FPKM (which we converted to TMP), and differential expression analysis was performed in R with DEseq2. For all RNA-seq data p-values refer to adjusted p-value (padj) calculated in DEseq2.

To find zone5 enriched transcription factors at each developmental stage we determined significantly differentially expressed transcription factors (from the Gene Ontology database annotation “DNA binding transcription factor activity”) between ventral and dorsal cells with a padj less than 0.05 and at least a twofold change in expression (see Table 1.) To get the most likely candidates driving zonal identity we further filtered the list for TFs with at least a 3-fold difference between dorsal and ventral cells, and an expression level of at least 15 TPM).

### Zonal vs non-zonal mOSN markers from olfactory lineage RNA-seq data:

To find ventrally enriched mOSN markers, we looked at non-OR genes differentially expressed between ventral mOSNs and dorsal or dorsomedial mOSNs (tomato-, GFP dim cells) with padj less than 0.05, and at least a twofold change in expression, of which there were 208; and performed the inverse analysis to generate a list of dorsal or dorsomedial enriched mOSN markers, of which there were 141 genes. To find non-zonal mOSN markers, we made a list of significantly upregulated genes (with a padj less than 0.05, and a fold change greater two) in mOSNs (tomato-, GFP dim cells) across all zones compared to iOSNs (tomato-, GFP+ bright cells) across all zones. We further filtered out genes that were significantly differentially expressed between ventral and dorsal or dorsomedial mOSNs and took the top 200 most significant genes.

### RNA-seq in ventral Nfi knockout mOSNs

To look at gene expression changes resulting from Nfi deletion in olfactory progenitors we used Nfi triple knockout (NFI A,B,X fl/fl, tdTomato, OMP-gfp, Krt5-CreER), AB only double knockout (NFI A,B fl/fl, tdTomato, OMP-gfp, Krt5-CreER), X only knockout (NFI X fl/fl, tdTomato, OMP-gfp, Krt5-CreER) or wt (tdTomato, OMP-gfp, Krt5-CreER) mice and followed the induction protocol for early knockout, described above. After rebuilding the MOE from knockout progenitors, we dissected ventral (zone5) MOE and FAC-sorted GFP+ mOSNs. RNA was extracted from sorted cells using Trizol and RNA-seq libraries were prepared with Nugen Nuquant RNA-seq library prep kit and sequenced 75PE on Nextseq 550. Reads were aligned exactly as described for zonal olfactory lineage data and similarly DEseq2 was used to determine differentially expressed genes between the different knockout and wt cells. To determine if ventral mOSN, dorsal mOSN and non-zonal mOSN markers change in ventral Nfi knockout cells, we analyzed the expression differences of the genes in our marker lists.

### Spatial Transcriptomics:

Whole Nfi ABX knockout and wt MOE were embedded in OCT and frozen on dry ice. 14μm cryosections of tissue were mounted onto Visium Spatial Gene Expression slides (10X Genomics) and kept at −80°C prior to processing. Tissue sections were fixed in methanol, stained with Hematoxylin and Eosin y, and imaged using a Nikon Eclipse Ti2 inverted microscope. Barcoded cDNA libraries of tissue sections were generated using the Spatial Gene Expression Reagent Kit (10X Genomics) according to the manufacture’s protocols. Libraries were sequenced on an Illumina NovaSeq instrument at the University of Chicago Genomics Core with the following runtype: 28 cycles (Read 1); 10 cycles (i7 index); 10 cycles (i5 index); 120 cycles (Read 2). Data were demultiplexed and processed using SpaceRanger v1.1.0. Reads were aligned to the mm10 2020-A reference mouse transcriptome (10X Genomics) and the OR transcriptome generated by Ibarra-Soria et al[58].

### Spatial Transcriptomics analysis:

Analysis was performed in R using STUtility[59]. Spatial spots expressing fewer than 2 OR genes and 3 OR transcripts were removed prior to analysis. Expression data across replicate sections were normalized using SCTransform, filtered to include only OR genes, and integrated using Harmony[60]. PCA was performed using the first 5 principal components and spatial spots were grouped into 5 clusters for both Nfi ABX knockout and wt samples. Heatmaps were generated using the top 20 highest expressed DEGs within each zone (Class I through Zone 5) for the wt sample and kept the same for the heatmap of the Nfi ABX knockout. The same top 20 DEGs for Zone 1, Zone 2, and Zone 5 were averaged per spot and overlaid against the H&E histology image (Fig 5 C). For zonal spot assignment (Fig 5 E), spots were designated to the zone with the largest summed normalized counts for all genes in that zone.

### Native chromatin immunoprecipitation from FAC-sorted cells:

Native chip was performed as described in detail [61]. Unless otherwise indicated all steps were carried out at 4°C. Briefly, FAC-sorted cells were pelleted at 600 rcf for 10 minutes in a swinging bucket centrifuge at 4°C and resuspended in cold Buffer I (0.3M Sucrose, 60 mM KCl, 15 mM NaCl, 5 mM MgCl_2_, 0.1 mM EGTA, 15 mM Tris-HCl pH 7.5, 0.1 mM PMSF, 0.5 mM DTT, 1x protease inhibitors). Cells were lysed by adding equal volume cold BufferII (Buffer I with 0.4% NP40) and incubating for 10 minutes on ice. Nuclei were pelleted 10 min at 1000 rcf and resuspended in 250ul cold MNase buffer (0.32M Sucrose, 4 mM MgCl_2_, 1 mM CaCl_2_, 50 mM Tris-HCl pH 7.5, 0.1 mM PMSF, 1x protease inhibitors). Micrococcal Nuclease digestion was carried out by adding 0.1U Micrococcal Nuclease (Sigma) per 100ul buffer and incubating for 1 min 40 sec in a 37°C water bath, then stopping the digestion by adding EDTA to a final concentration of 20mM. The first soluble chromatin fraction (S1) was collected by pelleting nuclei 10 min at 10,000 rcf at 4°C and taking the supernatant to store at 4°C overnight. Undigested, pelleted material was resuspended in 250ul cold Dialysis Buffer (1 mM Tris-HCl pH 7.5, 0.2 mM EDTA, 0.1 mM PMSF, 1x protease inhibitors) and rotating overnight at 4°C. The second soluble chromatin fraction (S2) was collected by pelleting insoluble material 10 min at 10,000 rcf at 4°C and taking the supernatant. S1 and S2 chromatin fractions were combined and used for immunoprecipitation with 5% material being retained for input. Equal cell numbers were used for control and knockout IPs, or between different cell types or zones. To perform immunoprecipitation (IP), chromatin was diluted to 1ml in Wash Buffer1 (50mM Tris-HCl pH 7.5, 10 mM EDTA, 125 mM NaCl, 0.1% Tween-20, 5 mM 1x protease inhibitors) and rotated overnight at 4°C with 1ug antibody. Dynabeads (10ul Protein A and 10ul Protein G per IP) were blocked overnight with 2 mg/ml yeast tRNA and 2 mg/mL BSA in Wash Buffer 1. Blocked beads were washed once with Wash Buffer 1, then added to antibody bound chromatin and rotated 2–3 hours at 4°C. Chromatin bound beads were washed 4x with Wash Buffer1, 3x with Wash Buffer 2 (50mM Tris-HCl pH 7.5, 10 mM EDTA, 175 mM NaCl, 0.1% NP40, 1x protease inhibitors), and 1x in TE pH 7.5. IP’d DNA was eluted by resuspending beads in 100 uL Native ChIP Elution Buffer (10 mM Tris-HCl pH7.5, 1 mM EDTA, 1% SDS, 0.1 M NaHCO_3_) in a thermomixer set to 37°C and 900 rpm for 15 minutes, repeating the elution 2x and combining the eluates. IPs and inputs (diluted to 200ul in elution buffer) were cleaned up with Zymo ChIP DNA columns (Zymo Research, D5205). Libraries were prepared with NuGEN Ovation V2 DNA-Seq Library Preparation Kit, and sequenced 50PE on HiSeq2500 and or 75PE on NextSeq 550.

### Native ChIP-seq analysis:

Sequenced reads were pre-processed by trimming adapters with Cutadapt, then aligned to the mm10 genome using Bowtie2, with default setting except for maximum insert size set to 1000 (-X 1000), allowing larger fragments to be mapped. Duplicate reads were removed with Picard, and high mapping quality reads were selected with Samtools (-q 30). After confirming replicates looked similar, they were merged with HOMER and used to generate signal tracks at 1bp resolution normalized to a library size of 10,000,000 reads. Signal density over OR genes was calculated with HOMER annotatePeaks.pl then normalized to the length of each OR gene. Native ChIP heatmaps were generated with deeptools with OR gene bodies re-scaled to 6kb and showing 2kb flanking on each side. To generate the heatmaps data from two nChIP replicates was merged, after confirming results from individual replicates looked similar.

### In situ Hi-C:

In situ Hi-C and library preparation was performed as exactly as described[14]. Briefly, FAC-sorted cells (inputs ranged from 150,000 to 500,000 cells) were pelleted at 500 rcf for 10 minutes and lysed in Lysis buffer (50 mM Tris pH 7.5 0.5% NP40, 0.25% sodium deoxychloate 0.1% SDS, 150 mM NaCl and 1x protease inhibitors) by rotating for 20 min at 4°C. Nuclei were pelleted at 2500 rcf, permabilized in 0.05% SDS for 20 min at 62 °C, then quenched in 1.1% Triton-X100 for 10 min at 37 °C. Nuclei were then digested with DpnII (6U/ul) in 1× DpnII buffer overnight at 37 °C. In the morning, nuclei were pelleted at 2,500*g* for 5 min and buffers and fresh DpnII enzyme were replenished to their original concentration and nuclei were digested for 2 additional hours. Restriction enzyme was inactivated by incubating 20 minutes at 62 °C. Digested ends were filled in for 1.5 hours at 37 °C using biotinylated dGTP. Ligation was performed for 4h at room temperature with rotation. Nuclei were pelleted and sonicated in 10 mM Tris pH 7.5, 1 mM EDTA, 0.25% SDS on a Covaris S220 (16 minutes, 2% duty cycle, 105 intensity, Power 1.8–1.85 W, 200 cycles per burst, max temperature 6°C). DNA was reverse crosslinked with RNAseA and Proteinase K overnight at 65 °C then purified with 2× Ampure beads following the standard protocol and eluted in water. Biotinylated fragments were enriched with Dynabeads MyOne Strepavidin T1 beads and on bead library preparation was carried out with NuGEN Ovation V2 DNA-Seq Library Preparation Kit, with some modifications: instead of heat inactivation following end repair beads were washed 2x for 2 min at 55 °C with Tween Washing Buffer (TWB)(0.05% Tween, 1 M NaCl in TE pH 7.5) and 2x with 10 mM Tris pH 7.5 to remove excess detergent. After ligation of adapters beads were washed 5x with TWB and 2x with 10 mM Tris pH 7.5. Libraries were amplified for 10 cycles and cleaned up with 0.8V Ampure beads. Each experiment was performed with two biological replicates and prepared Hi-C libraries were sequenced 75PE on NextSeq 500.

### In situ Hi-C analysis:

Reads were aligned to the mm10 genome using the distiller pipeline (https://github.com/mirnylab/distiller-nf), uniquely mapped reads (mapq > 30) were retained and duplicate reads discarded. Contacts were then binned into matrices using cooler. [62]. Analysis was performed on data pooled from two biological replicates, after confirming that the results of analysis of individual replicates were similar. Hi-C contact maps of OR clusters on chromosome 2 were generated with raw counts of Hi-C contacts normalized to counts/billion at 100kb resolution. The maximum value on the color scale was set to 150 contacts per 100kb bin. Analysis of zonal OR gene cluster contacts was performed normalized counts binned at 50kb resolution. All analyses were repeated using balanced counts generated by cooler (-mad-max 7), with similar results except balanced matrices discarded almost 10% of OR cluster bins due to relatively poor sequencing coverage.

### Dip-C:

To isolate mature olfactory sensory neurons, Castaneous (Cas) mice were crossed to OMP-ires-GPF mice. MOE was collected from adult heterozygous mice resulting from this cross. The tissue was dissected into zone 1 and zone4/5, fixed for 10 minutes in 2% formaldehyde and FAC-sorted to isolate GFP+ mOSNs. Dip-C was performed as described[37] on 96 mature OSNs: 48 each from dorsal and ventral MOE. Briefly, cells were lysed in Hi-C Lysis Buffer (10mM Tris pH8, 10mM NaCl, 0.2% NP40, 1x protease inhibitors) on ice for 15 minutes, nuclei were pelleted at 2500 rcf for 5 min at 4°C, then resuspended in 0.5% SDS and permeabilized 10 minutes at 62 °C then quenched in 1.1% Triton X-100 15min at 37 °C. Nuclei were digested in 1x DpnII buffer and 6U/ul DpnII enzyme and digested overnight at 37°C. Nuclei were then washed once in Ligation Buffer, and resuspended in Ligation buffer with 10U T4 DNA Ligase (Life Tech), and incubated for 4 hours at 16oC shaking at 600rpm. After ligation nuclei were pelleted and resuspended in cold PBS with DAPI to a final concentration of 300nM and GFP+ cells were FAC-sorted into a 96 well plate with 2ul lysis buffer (20mM Tris pH 8, 20mM NaCl, 0.15% Triton X-100, 25mM DTT, 1mM EDTA, 500nM Carrier ssDNA, and 15ug/mL Qiagen Protease) and lysed for 1 hour at 50°C and inactivated 15 minutes at 70°C. DNA was transposed by adding 8ul transposition buffer (12.5 mM TAPS pH 8.5, 6.25mM MgCl2, 10% PEG 8000) with ~0.0125 uLTn5 (Vanzyme) and incubated at 55°C for 10 min, then stopped with transposome removal buffer (300nM NaCl, 45 mM EDTA, 0.01% Triton X-100 with 100ug/mL Qiagen Protease) and incubated at 50°C for 40 minutes and 70°C for 20 minutes. Libraries were amplified 14 cycles with i5 and i7 Nextera primers, with unique barcodes for each cell. Libraries from all cells were pooled and cleaned up with Zymo DNA Clean and Concentrate Kit. Libraries were sequenced 150PE on NextSeq 550.

### Dip-C analysis:

Sequenced Dip-C reads were processed according to the Dip-C pipeline (https://github.com/tanlongzhi/dip-c). Reads were aligned to mm10 with BWA mem, and hickit was used to determine the haplotype of each contact based on SNPs between Cas and OMP-ires-GFP mice and make a model of the 3D genome. Since OMP-ires-GFP mice were a mixture of Bl6/129 strains, we only included SNPs that were unique to Cas mice to distinguish homologs. After alignment, cells were filtered using several quality control metrics described in Tan et al. 2019: We excluded cells that had less than 20,000 reads, cells that had a low contact-to-read ratio, and cells that had a high variability in 3D structure across computational replicates. Only 4 of 96 cells failed these metrics. Overall, the median number of contacts across cells was over 400,000. Computational analysis OR genes and Greek Island enhancers, including computing average contact densities and analysis of the 3D models, was performed using the Dip-C pipeline. Average contact densities between OR genes and/or Greek Islands were calculated with ‘dip-c ard’. Pairwise distances between OR genes and/or Greek Islands from the 3D models were extracted with ‘dip-c pd’. Heatmaps of pairwise distance were either ordered by genomic position or reordered using hierarchical clustering. To determine the size of OR gene aggregates and Greek Island hubs, the number of OR genes or Greek Islands within a specified radius of was calculated with ‘network_around.py’. 3D models were visualized with PyMol and used to generate sequential slices of the nucleus.

### Spatial Transcriptomics:

### Nfi ABX knockout and wt MOE were embedded in OCT.

### Antibodies:

Olfr17 antibody were raised in rabbits against epitope RRIIHRTLGPQKL located at the C-terminus of the OR protein. Olfr1507 antibody was described in [63]. The following antibodies were used for native ChIP: H3K79me3 (abcam ab2621) and H3K9me3 (ab8898).

## Supplementary Material

Supplement 1

## Data Availability

Sequencing data (RNA-seq, scRNA-seq, ChIP-seq) reported in this paper are publicly available at GEO under accession number 158730. HiC and Dip-C data are publicly available at the 4DN Data Portal (https://data.4dnucleome.org/). This paper also makes use of published Olfr17+ mOSN Hi-C data available at the 4DN Data Portal under the accession number 4DNESNYBDSLY.
