# Supplementary figures and images for: Opposing, spatially-determined epigenetic forces impose restrictions on stochastic olfactory receptor choice
Source: bioRxiv. 2023 Sep 19:2023.03.15.532726. Originally published 2023 Mar 15. Preprint. [Version 2] doi: 10.1101/2023.03.15.532726 (PMC10055043; doi:10.1101/2023.03.15.532726)

Supplementary Figure S1

A.

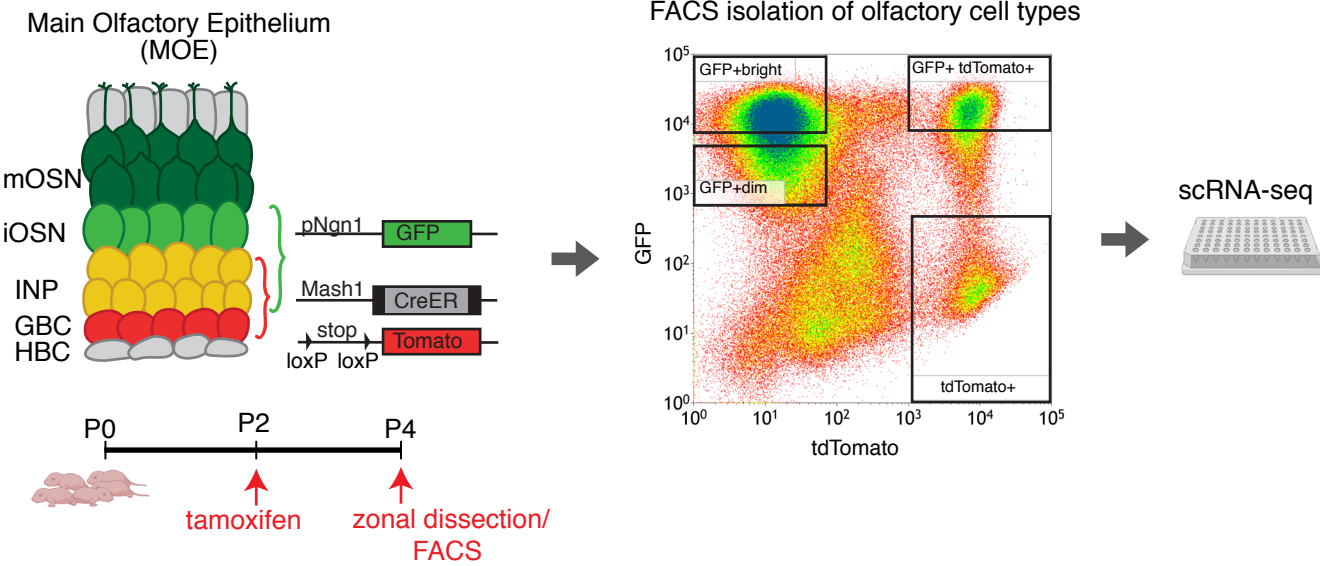

B.

scRNA-seq clustering

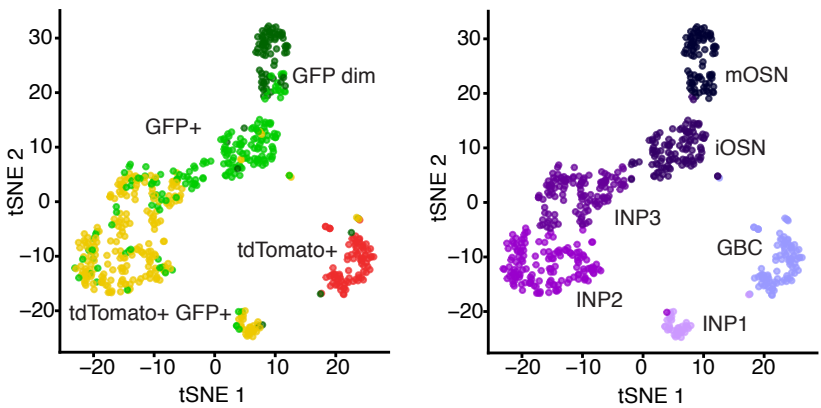

C.

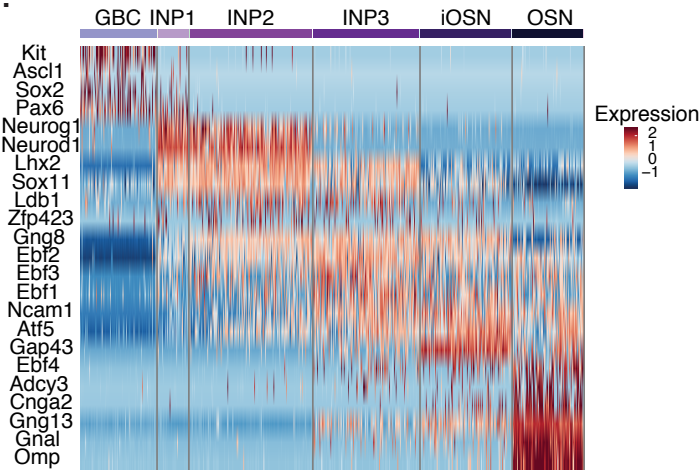

Supplement: Supplement 1 — Supplementary Figure S1 (related to Figure 1): Experimental strategy for isolating cells at different stages of olfactory sensory neuron development for single cell RNA-seq. (A) Genetic and experimental strategy for isolating four cell populations at different stages of olfactory sensory neuron development (GBCs, INPs, iOSNs and mOSNs) from the same tissue. A representative FACS plot is shown. GBC, globose basal cell; INP, immediate neuronal precursor; iOSN, immature olfactory sensory neurons; mOSN, mature olfactory sensory neuron. (B) t-SNE plots of clustering of FAC-sorted cell populations with Seurat based on the most variable genes showing the separation of single cells into 6 populations. Plots show the relationship between the FAC-sorted populations (left) and cell lineage (right). (C) The 6 populations in (B) were assigned cell identities based on expression of known MOE markers, whose expression is shown in the heatmap. Expression for each gene is represented in terms of log2 fold change relative to its average expression. [file media-1.pdf]

Supplementary Figure S2

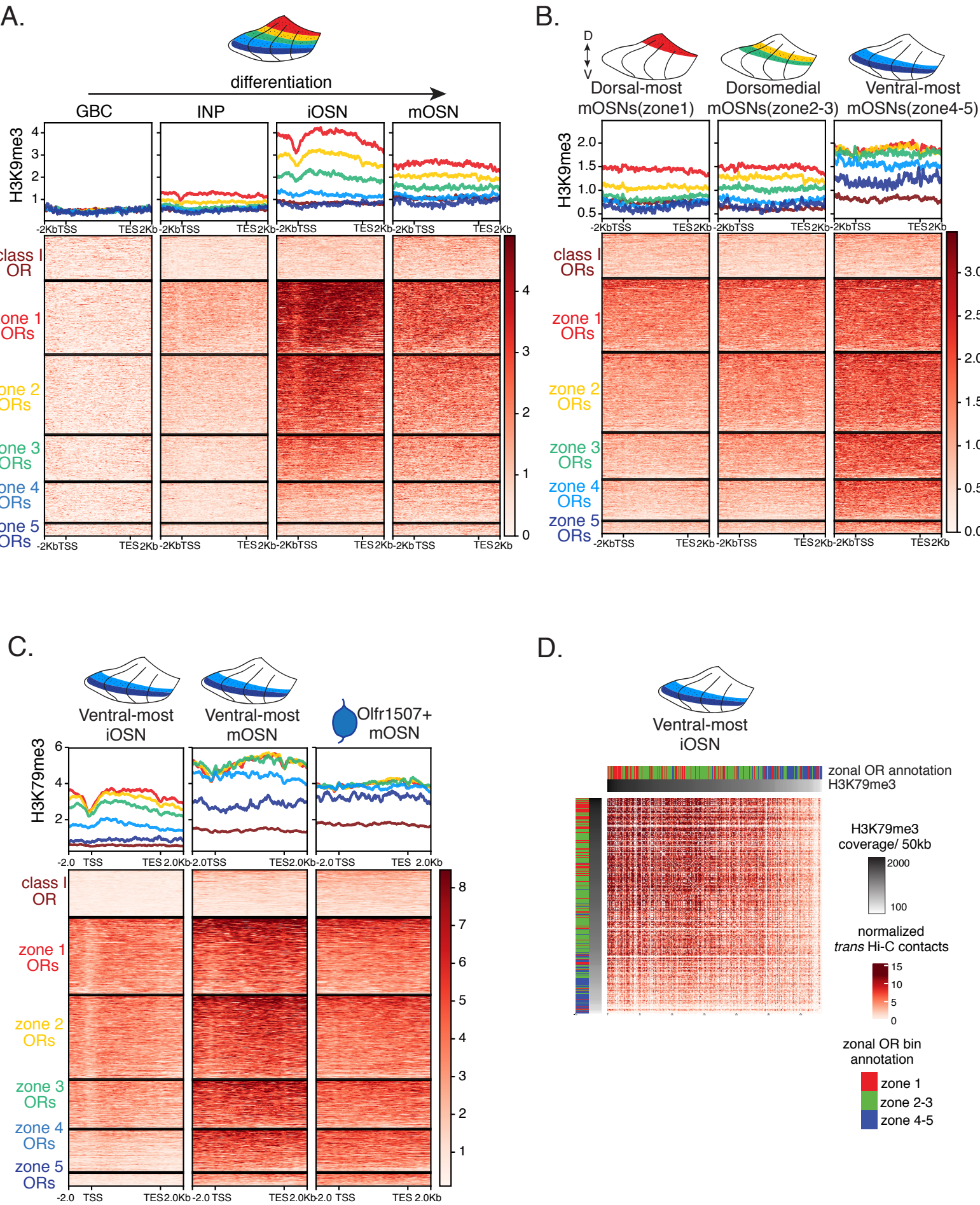

Supplement: Supplement 2 — Supplementary Figure S2 (related to Figure 2): Progressive accumulation of heterochromatin on OR genes in space and time. (A) H3K9me3 native ChIP seq in GBC, INP, iOSN and mOSN populations shows a similar onset of deposition to that of H3K79me3. Each row of the heatmaps shows coverage over an OR gene body (scaled to 6kb with 2kb flanking on either side), separated into categories by their zonal identity. (B) H3K9me3 native ChIP-seq in mOSNs from zonally dissected MOE. Colored schematics above each heatmap depict the zone of dissection. (C) H3K79me3 native ChIP-seq in cells at different developmental stages from ventral-most dissected MOE: iOSN (left), mOSN (middle), and a pure population of Olfr1507-expressing mOSNs (a zone5 OR) (right). H3K79me3 heterochromatin is absent from zone5 ORs in ventral-most iOSNs and is deposited progressively as the cells mature. (D) Increased H3K79me3 deposition is correlated with increased interchromosomal interactions between OR gene loci. Heatmap of normalized interchromosomal Hi-C contacts between OR cluster bins. Each bin is ordered by H3K79me3 ChIP signal (gray color bar on top) and annotated according to the zonal identity of its resident OR genes: zone1 ORs, red; zone2–3 ORs, green; zone4–5 ORs, blue. Class I OR genes (which are also expressed in zone1) make few interchromosomal interactions throughout all zones of the MOE and were thus excluded from this analysis. OR cluster regions that have higher levels of H3K79me3 and are enriched for zone1 ORs have increased trans Hi-C contacts. [file media-2.pdf]

# Supplementary Figure S4

A.

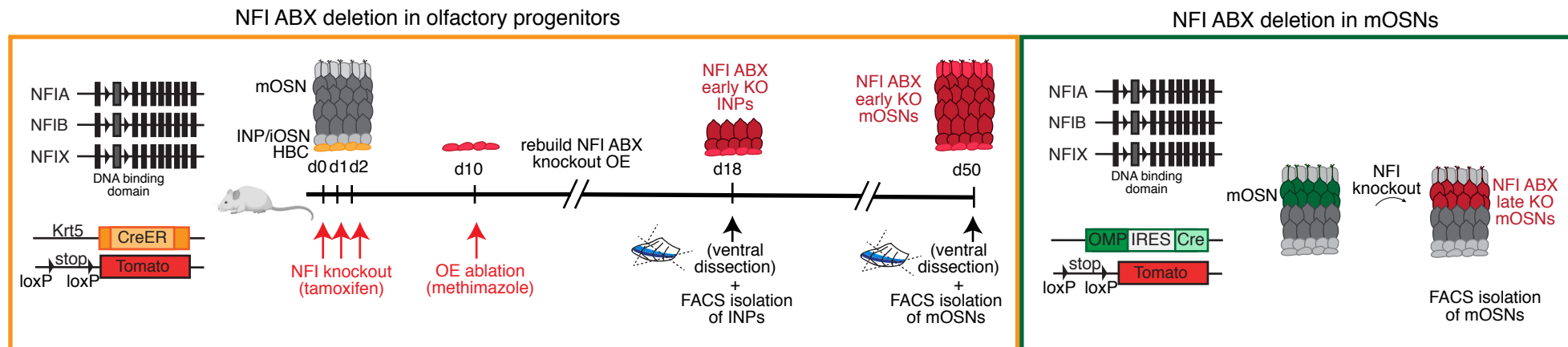

B.

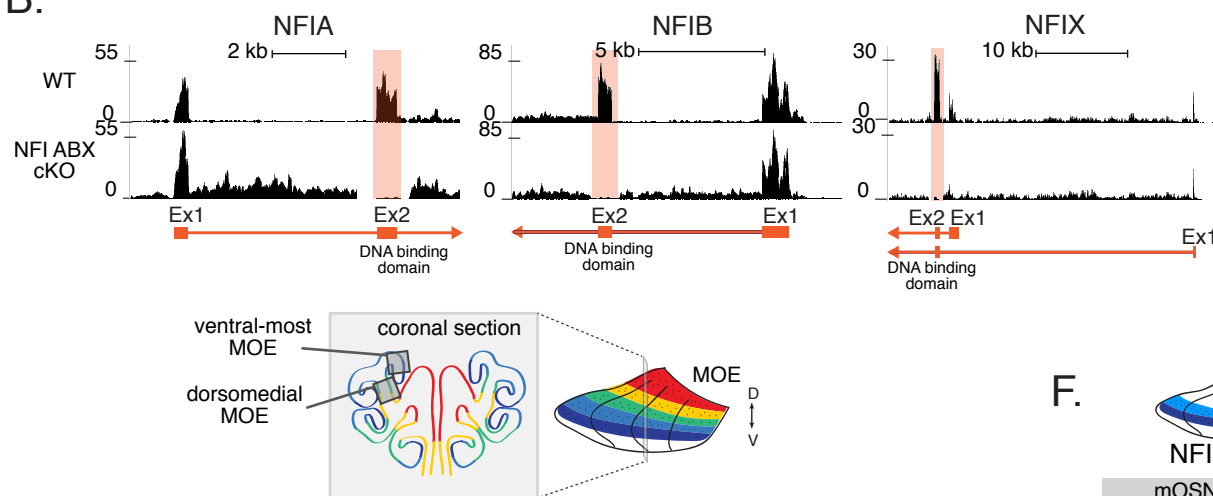

C.

Differentially expressed ORs in NFI cKO zone5 OSNs

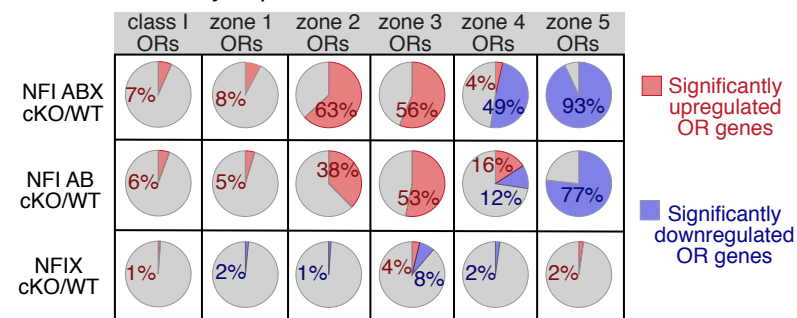

F.

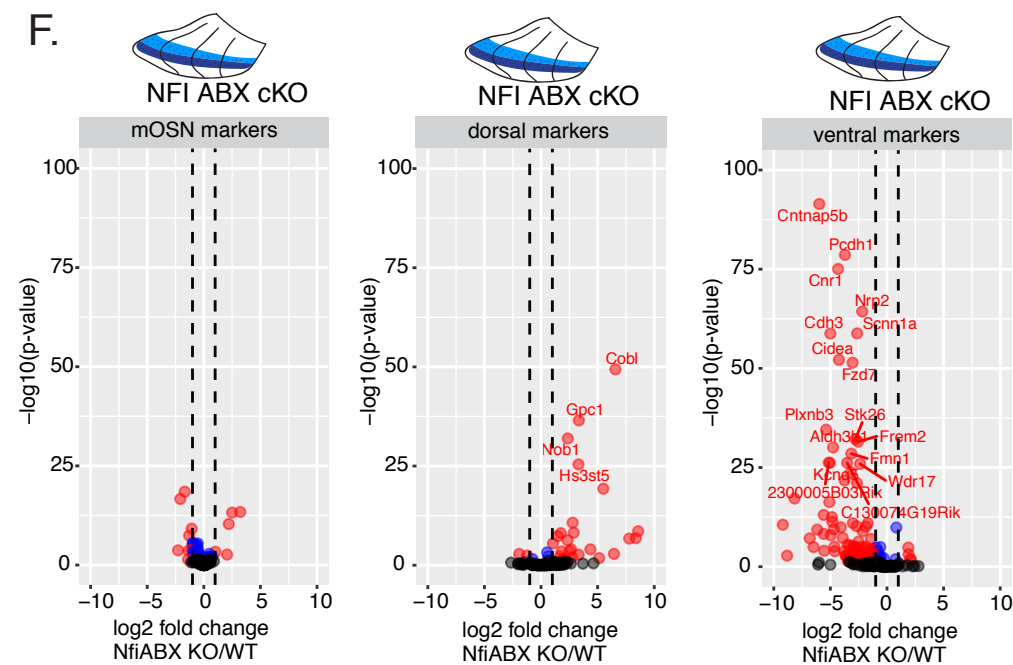

D.

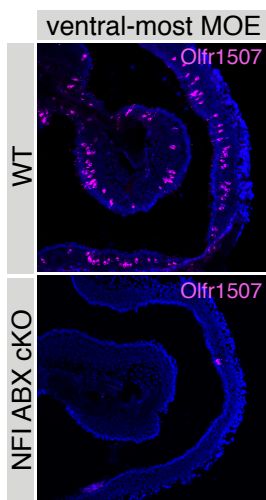

E.

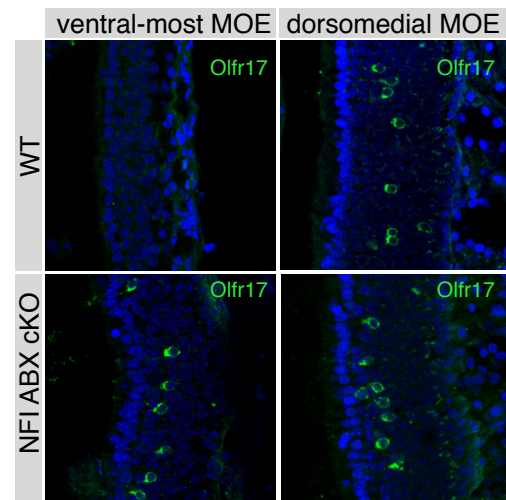

Supplement: Supplement 4 — Supplementary Figure S4 (related to Figure 4): NFI ABX deletion in zone5 olfactory epithelium results in a shift in the OR repertoire. (A) Schematic showing the experimental and genetic strategy for deleting NFI transcription factors in olfactory progenitor HBC cells using the Krt5-CreER driver (left), and in mOSNs using the OMP-IRES-Cre driver (right). Upon induction of NFI deletion in HBCs with tamoxifen, we chemically ablate the MOE with methimazole, allowing the quiescent HBCs to rebuild the epithelium. After 40 days, OSNs that were produced from the triple NFI conditional knockout (NFI ABX cKO) or control HBCs will be marked with a tomato reporter, and isolated by FACS. (B) Olfr1507 (a zone 5 identity OR) immunofluorescence (magenta) in MOE sections of adult NFI ABX triple cKO mice and age-matched control (wt) mice. Nuclei are stained with Dapi (blue). Images were taken in the same ventral location, indicated on the schematic of a coronal section of the MOE. (C) Olfr17 (a zone 2 identity OR) immunofluorescence (green) in MOE sections of adult NFI ABX triple cKO mice and age-matched control mice (wt), shows normal Olfr17 expression in dorsomedial MOE and ectopic spreading of Olfr17 expression into more ventral zones. Images were taken in the same spots in the MOE, indicated in the schematic. Nuclei are stained with Dapi (blue). (D) Differential expression analysis of OR genes in the different NFI cKO genotypes. Percentages of significantly upregulated ORs (red) and downregulated ORs (blue) are shown. (E) Volcano plot showing expression of non-zonal mOSN markers, dorsal mOSN markers and ventral mOSN markers in ventral mOSNs isolated from NFI ABX triple cKO relative to wt MOE. Only 13/200 non-zonal mOSN markers are significantly downregulated in NFI ABX triple knockout, compared to 90/207 ventral markers significantly downregulated and 29/138 dorsal markers that were significantly upregulated. Blue: significantly differentially expressed genes (p-value < 0.5), red [file media-4.pdf]

Supplementary Figure S5

A.

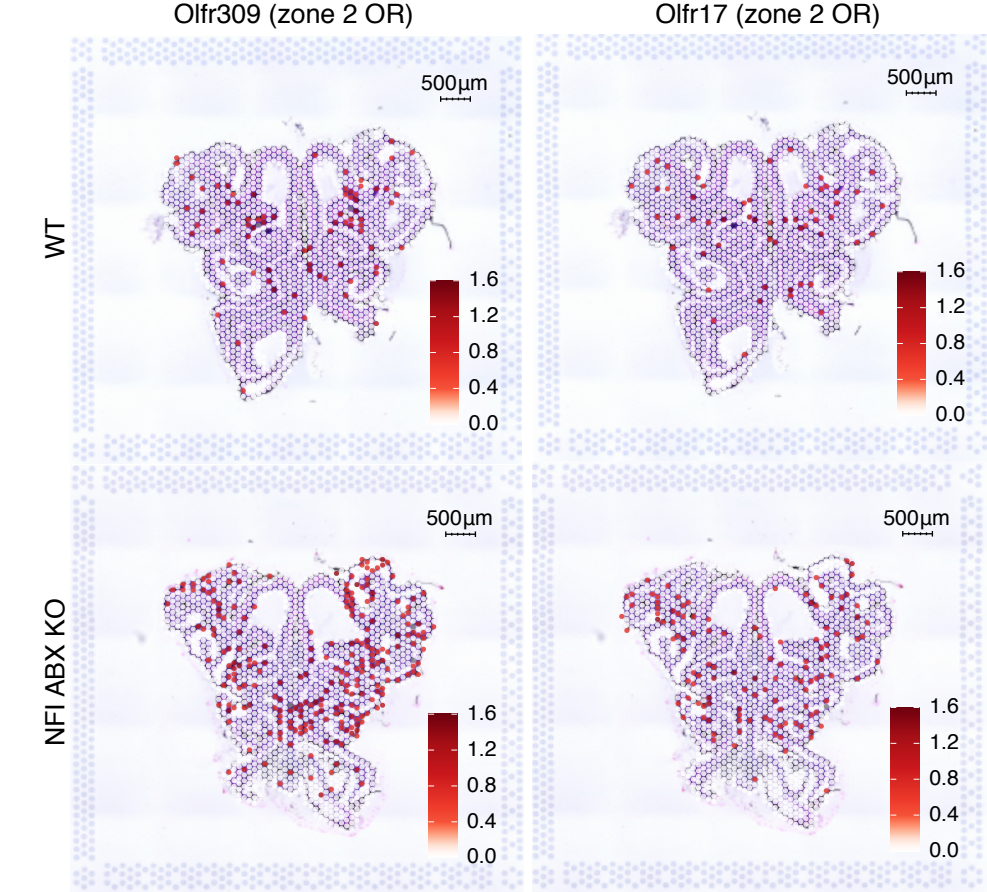

B.

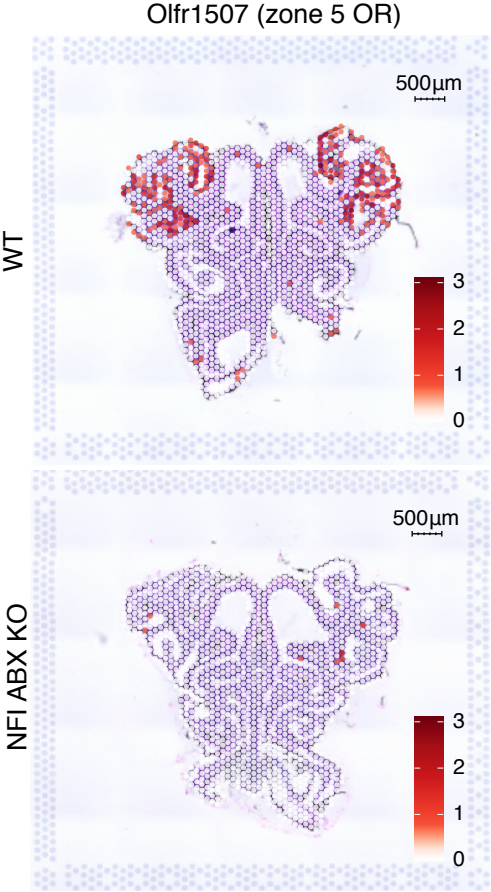

Supplement: Supplement 5 — Supplementary Figure S5 (related to Figure 5): Expression of zone2 ORs genes spreads ventrally in NFI ABX knockout MOE. (A) Normalized expression per spatial spot of the zone 2 identity OR genes Olfr309 and Olfr17 overlaid against histological H&E tissue image of wt control and NFI ABX knockout MOE. Olfr309 is the highest expressed zone2 OR gene in the control dataset. Expression of Olfr309 and Olfr17 in the NFI ABX knockout sample spreads to more ventral areas of the MOE compared to the control. (B) Normalized expression per spatial spot of Olfr1507, the most highly expressed zone 5 OR gene in the control MOE dataset. As expected, Olfr1507 is expressed in the ventral zone 5 anatomical region in the control MOE, while its expression is almost completely absent in the NFI ABX knockout MOE. [file media-5.pdf]

Supplementary Figure S6

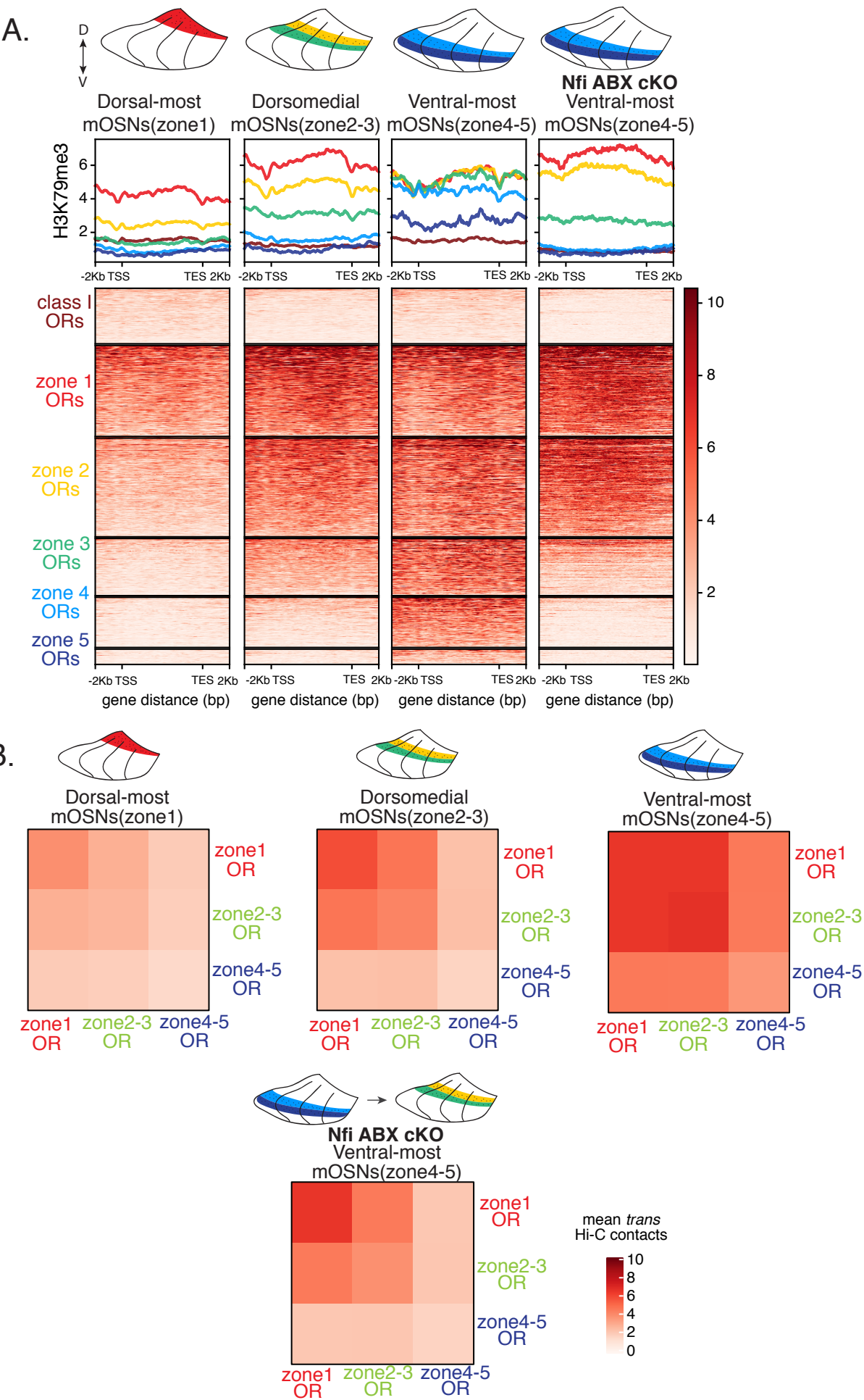

Supplement: Supplement 6 — Supplementary Figure S6 (related to Figure 6): Ventral NFI ABX cKO cells closely resemble dorsomedial cells in chromatin state and compartment formation. (A) Heatmap with side-by-side comparison of H3K79me3 native ChIP-seq signal over OR genes in wt control dorsal, dorsomedial, and ventral mOSNs compared to ventral NFI ABX cKO mOSNs. Ventral NFI ABX cKO mOSNs have a chromatin state most similar to that of wt control dorsomedial mOSNs. (B) Side-by-side heatmaps of average interchromosomal Hi-C contacts between OR genes of different zonal identities show the nuclear OR gene interactome in ventral NFI ABX cKO OSNs is most similar to that of wt control dorsomedial mOSNs, especially regarding the intermediate Hi-C contact frequency of zone 2–3 identity ORs. [file media-6.pdf]

# Supplementary Figure S7

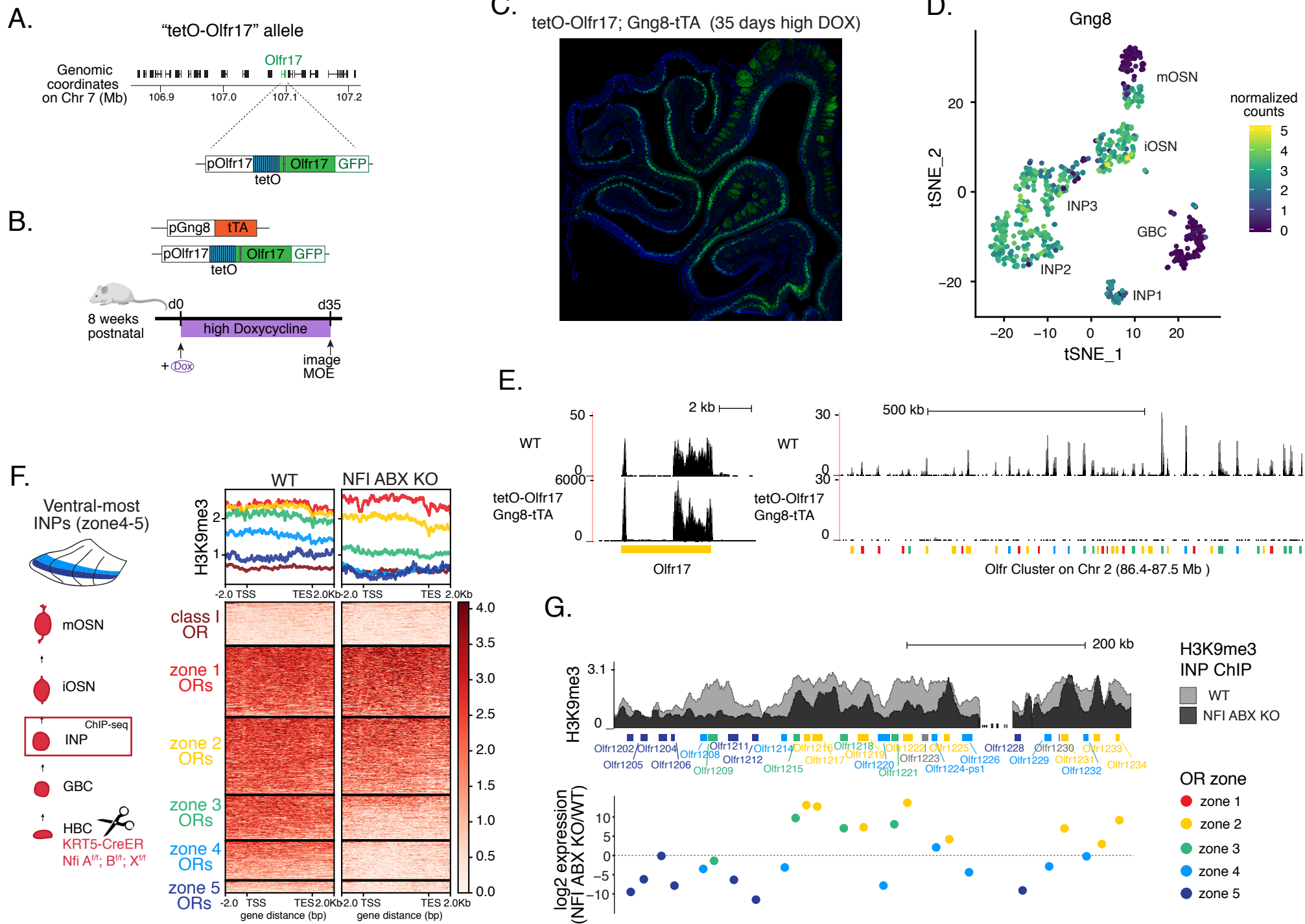

Supplement: Supplement 7 — Supplementary Figure S7 (related to Figure 7): Hijacking OR gene choice by inducing OR transcription at the stage of polygenic OR transcription. (A) Schematic showing that the tetO promoter and the IRES-GFP tag have been inserted in the endogenous Olfr17 gene locus on Chromosome 7 (top). Olfr17 (green) resides in a large OR gene cluster surrounded by other OR genes (shown in black). Early induction of this “tetO-Olfr17” allele in olfactory progenitors with the Gng8-tTA driver results in sustained, tTA-independent, expression in mature OSNs. GFP signal from induced tetO-Olfr17 expression persists in most OSNs after 35 days in high doxycycline diet (B,C). (B-C) Schematic of the high doxycycline feeding protocol used to inhibit the tTA and thereby confirm tTA-independent tetO-Olfr17 expression in mOSNs (B). After adult (> 8 weeks old) tetO-Olf17 with Gng8-tTA mice were placed in high DOX diet for 35 days their OSNs continued to express GFP (C), excluding the possibility that tTA traces could support tetO-Olfr17 transcription. Note the distinction from the low DOX treatment described in Figure 7, where mice were exposed in utero to doxycycline and never had a chance to induce tetO-Olfr17 expression at high levels. (D) Feature plot showing of Gng8 expression in single cell RNA-seq from sorted cells in the MOE (as described in Supplementary Figure S1). Clustering shows 6 populations corresponding to different stages of mOSN development, with Gng8 being expressed in INP and iOSN cell types but not in mOSNs, consistent with previous reports. (E) Genome track of RNA-seq in tetO-Olfr17+ OSNs from Gng8-tTA; tetO-Olfr17 mice and previously published WT OSNs (GSE112153). Cells expressing the INP/iOSN induced tetO-Olfr17 allele do not express any other OR genes. (F, G) H3K9me3 native ChIP in ventral INP cells isolated from NFI ABX KO and age-matched WT control mice (as described in Supplementary Figure S4) shows heterochromatin deposition at the onset of OR choice. Heatmap of ChI [file media-7.pdf]
