# Supplementary material for: Opposing, spatially-determined epigenetic forces impose restrictions on stochastic olfactory receptor choice
Source: bioRxiv. 2023 Sep 19:2023.03.15.532726. Originally published 2023 Mar 15. Preprint. [Version 2] doi: 10.1101/2023.03.15.532726 (PMC10055043; doi:10.1101/2023.03.15.532726)

Supplementary Figure 3

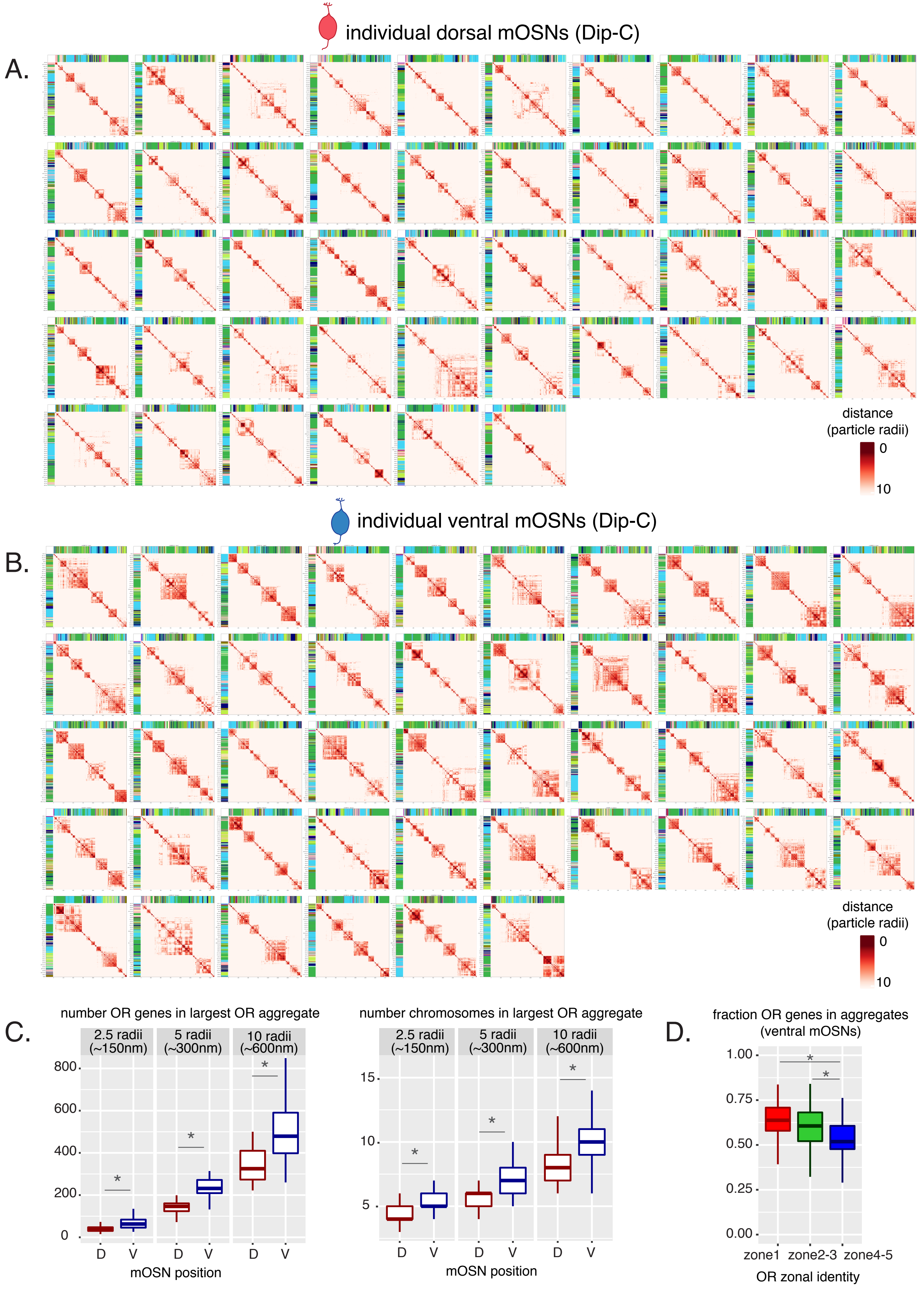

Supplement: Supplement 3 — Supplementary Figure S3 (related to Figure 3): OR compartments are highly variable between single cells but show a consistent difference between cells of different zones. (A, B) Heatmaps of distances between OR genes computed from Dip-C 3D genome structures show OR genes within 10 particle radii (~600nm) in each dorsal mOSN (A) and ventral mOSN (B). Heatmaps are hierarchically clustered to show intrachromosomal OR gene aggregates. (C) Analysis of all OR genes within either 2.5, 5 or 10 particle radii (analogous to ~150, 300 and 600nm) of one another in 3D nuclear structures shows that OR genes in ventral mOSNs form larger (composed of more OR genes) (left) and more complex (composed of ORs residing on more chromosomes) (right) OR compartments. D = dorsal, V = ventral. Asterisk denotes Wilcoxon rank sum test p-value <0.001). (D) In each ventral mOSN a significantly smaller fraction of ventral identity (zone 4–5) OR genes are in proximity of ORs from other chromosomes than dorsomedial (zone 2–3) or dorsal (zone 1) identity ORs. Proximity is defined as within 2.5 particle radii (analogous to ~150 nm) in 3D nuclear structures. Asterisk denotes Wilcoxon rank sum test p-value <0.01). [file media-3.pdf]
