# Supplementary material for: Opposing, spatially-determined epigenetic forces impose restrictions on stochastic olfactory receptor choice
Source: bioRxiv. 2023 Mar 15:2023.03.15.532726. Preprint. [Version 1] doi: 10.1101/2023.03.15.532726 (PMC10055043; doi:10.1101/2023.03.15.532726)

bioRxiv preprint doi: https://doi.org/10.1101/2023.03.15.532726; this version posted March 15, 2023. The copyright holder for this preprint (which was not certified by peer review) is the author/funder, who has granted bioRxiv a license to display the preprint in perpetuity. It is made available under aCC-BY 4.0 International license.

Supplemental Figures and Table Legends

Supplementary Figure S1 (related to Figure 1): Experimental strategy for isolating cells at different stages of olfactory sensory neuron development for single cell RNA-seq.

(A) Genetic and experimental strategy for isolating four cell populations at different stages of olfactory sensory neuron development (GBCs, INPs, iOSNs and mOSNs) from the same tissue. A representative FACS plot is shown. GBC, globose basal cell; INP, immediate neuronal precursor; iOSN, immature olfactory sensory neurons; mOSN, mature olfactory sensory neuron.
(B) t-SNE plots of clustering of FAC-sorted cell populations with Seurat based on the most variable genes showing the separation of single cells into 6 populations. Plots show the relationship between the FAC-sorted populations (left) and cell lineage (right).
(C) The 6 populations in (C) were assigned cell identities using expression of known MOE markers, whose expression is shown in the heatmap. Expression for each gene is represented in terms of log2 fold change relative to its average expression.

Supplementary Figure S2 (related to Figure 2): Progressive accumulation of heterochromatin on OR genes in space and time.
(A) H3K9me3 native ChIP seq in GBC, INP, iOSN and mOSN populations shows a similar onset of deposition to that of H3K79me3. Each row of the heatmaps shows coverage over an OR gene body (separated into categories by their zonal index).
(B) H3K9me3 native ChIP-seq in mOSNs from zonally dissected MOE. Colored schematics above each heatmap depicts the zone of dissection.
(C) H3K79me3 native ChIP-seq in cells at different developmental stages from ventral-most dissected MOE: iOSN (left), mOSN (middle), and a pure population of Olfr1507-expressing OSNs (a zone5 OR) (right). H3K79me3 heterochromatin is absent from zone5 ORs in ventral-most iOSNs and is deposited progressively as the cells mature.
(D) Increased H3K79me3 deposition is correlated with increased interchromosomal interactions between OR gene loci. Heatmap of normalized interchromosomal Hi-C contacts between OR cluster bins. Each bin is ordered by H3K79me3 ChIP signal (gray color bar on top) and annotated according to the zonal index of their resident OR genes: zone1 ORs, red; zone2-3 ORs, green; zone4-5 ORs, blue. OR cluster regions that have higher levels of H3K79me3 and are enriched for zone1 ORs have increased *trans* Hi-C contacts.

bioRxiv preprint doi: https://doi.org/10.1101/2023.03.15.532726; this version posted March 15, 2023. The copyright holder for this preprint (which was not certified by peer review) is the author/funder, who has granted bioRxiv a license to display the preprint in perpetuity. It is made available under aCC-BY 4.0 International license.

Supplementary Figure S3 (related to Figure 3): OR compartments are highly variable between single cells but show a consistent difference between cells of different zones.
(A, B) Heatmaps of distances between OR genes computed from Dip-C 3D genome structures show OR genes within 10 particle radii (~600nm) in each dorsal mOSN (A) and ventral mOSN (B). Heatmaps are hierarchically clustered to show intrachromosomal OR gene aggregates.

Supplementary Figure S4 (related to Figure 4): Nfi ABX deletion in zone5 olfactory epithelium results in a shift in the OR repertoire.
(A) Schematic showing the experimental and genetic strategy for deleting Nfi transcription factors in olfactory progenitor HBC cells using a KRT5-CreER driver (left), and mOSNs using an Omp-IRES-Cre driver (right). Upon induction of Nfi deletion in HBCs with tamoxifen, we chemically ablate the MOE with methimazole, allowing the quiescent HBCs to rebuild the epithelium. After 50 days, OSNs that were produced from the Nfi cKO/control HBCs will be marked red, and isolated by FACS, with the use of a second GFP reporter that marks each OSN differentiation stage.
(B) Olfr1507 (a zone5 OR) immunofluorescence (magenta) in MOE sections of adult Nfi ABX triple cKO mice and littermate control (wt) mice. Nuclei are stained with Dapi (blue). Images were taken in the same ventral location, indicated on the schematic of a coronal section of the MOE.
(C) Olfr17 (a zone2 OR) immunofluorescence (green) in MOE sections of adult NFI ABX triple cKO mice and littermate control mice (wt), shows normal Olfr17 in dorsomedial MOE and ectopic spreading of Olfr17 expression into more ventral zones. Images were taken in the same spots in the MOE, indicated in the schematic. Nuclei are stained with Dapi (blue).
(D) Differential expression analysis of OR genes in the different NFI cKO genotypes. Percentages of significantly upregulated ORs (red) and downregulated ORs (blue) are shown.
(E) Volcano plot showing expression of non-zonal mOSN markers, dorsal mOSN markers and ventral mOSN markers in ventral mOSNs isolated from NFI ABX triple cKO relative to wt MOE. Only 13/200 non-zonal mature markers are significantly downregulated in NFI ABX triple knockout, compared to 90/207 ventral markers significantly downregulated and 29/138 dorsal markers that were significantly upregulated. Blue: significantly differentially expressed genes (p-value < 0.5), red: significantly differentially expressed genes with a > 2-fold change.

Supplementary Figure S5 (related to Figure 5): Expression of zone2 ORs genes spreads ventrally in Nfi ABX knockout MOE.
(A) Normalized expression per spatial spot of the zone2 index ORs Olfr309 and Olfr17 overlaid against histological H&E tissue image of wt and Nfi ABX knockout MOE. Olfr309 is the highest expressed zone2 OR gene in the wt dataset. Compared to wt, expression of Olfr309 and Olfr17 in the Nfi ABX knockout sample is spread to more ventral areas of the MOE.
(B) Normalized expression per spatial spot of the Olfr1507, the most highly expressed zone5 OR gene in the wt MOE dataset. As expected, Olfr1507 is expressed in the ventral zone 5 anatomical region in the wt dataset. Expression of Olfr1507 in the Nfi ABX knockout is lower than in wt but is localized to the same region of the MOE.

Supplementary Figure S6 (related to Figure 6): Ventral Nfi ABX cKO cells closely resemble dorsomedial cells in chromatin state and compartment formation.
(A) Heatmap with side-by-side comparison of H3K79me3 ChIP-seq signal over OR genes in control (wt) dorsal, dorsomedial, and ventral mOSNs compared to ventral Nfi ABX cKO mOSNs. Ventral Nfi ABX cKO mOSNs have a chromatin state most similar to that of control (wt) dorsomedial mOSNs.
(B) Side by side heatmaps of average interchromosomal Hi-C contacts between OR genes of different zonal indexes show the nuclear OR interactome in ventral Nfi ABX cKO OSNs is most similar to that of control (wt) dorso-medial mOSNs, especially in regards of the intermediate HiC contacts of the zone 2-3 ORs.

Supplementary Figure S7 (related to Figure 7): Hijacking OR gene choice by inducing OR transcription at the stage of polygenic OR transcription.
(A) Schematic showing that the tetO promoter and the iresGFP tag have been inserted in the endogenous Olfr17 gene locus in Chromosome 7 (top). Olfr17 (red line) resides in a large OR gene cluster and is surrounded by other OR genes, depicted by black lines. Early induction of tetO-Olfr17iresGFP by Gng8-tTA results in tTA-

bioRxiv preprint doi: https://doi.org/10.1101/2023.03.15.532726; this version posted March 15, 2023. The copyright holder for this preprint (which was not certified by peer review) is the author/funder, who has granted bioRxiv a license to display the preprint in perpetuity. It is made available under aCC-BY 4.0 International license.

independent expression as GFP signal is expressed most OSNs 35 days after adult mice were placed in high doxycycline diet (bottom).

(B-C) Schematic of the high doxycycline feeding protocol used to confirm tTA-independent tetO-Olfr17iresGFP expression in mOSNs (B). Adult mice (8week old mice) were placed in high-dox diet for 35 days, and their OSNs continue to express GFP (C), excluding the possibility that tTA traces could support Olfr17 transcription. Note the distinction from the low-dox diet described in Figure 7, where mice were exposed *in utero* to doxycycline and never had a chance to induce Olfr17 expression at high levels.

(D) t-SNE clustering of single cells into 6 populations (as described in Figure S1) shows Gng8 expression in INP and iOSN cell types but not in mOSNs, consistent with previous reports.

(E, F) H3K9me3 native ChIP in ventral INP cells isolated from Nfi ABX cKO and control (wt) littermate mice (as described in Figure S4). Heatmap of ChIP signal (E) shows decreased heterochromatin on “in zone” OR genes at the onset OR choice in control (zone 4-5 ORs) and triple Nfi cKO (zone 2-3) INPs. Overlay of ChIP signal tracks of over an OR cluster (D, top) shows variable changes between control (wt) and Nfi ABX cKO heterochromatin levels in INPs throughout the cluster. OR genes are colored according to their zonal index. Log2 fold change in expression between ventral Nfi ABX knockout and control (wt) mOSNs for each OR in the cluster (D, bottom). Note that zone 2 OR genes ectopically chosen in ventral cKO OSNs have decreased heterochromatin in ventral cKO INPs.

Supplemental Table 1 (related to Figure 4): Complete list of transcription factors differentially expressed between dorsal and ventral cells at various stages of OSN differentiation. In the main Figure 4 we only included TFs with a 3 fold difference between the two zones but here we have a less stringent list including TFs with 2 fold differential expression.

Supplemental Table 2 (related to STAR methods): Complete list of mouse genotypes analyzed in this manuscript.

Supplemental Table 3 (related to STAR methods): List of reagents used for spatial transcriptomics.

bioRxiv preprint doi: https://doi.org/10.1101/2023.03.15.532726; this version posted March 15, 2023. The copyright holder for this preprint (which was not certified by peer review) is the author/funder, who has granted bioRxiv a license to display the preprint in perpetuity. It is made available under aCC-BY 4.0 International license.

Supplemental Figure S1

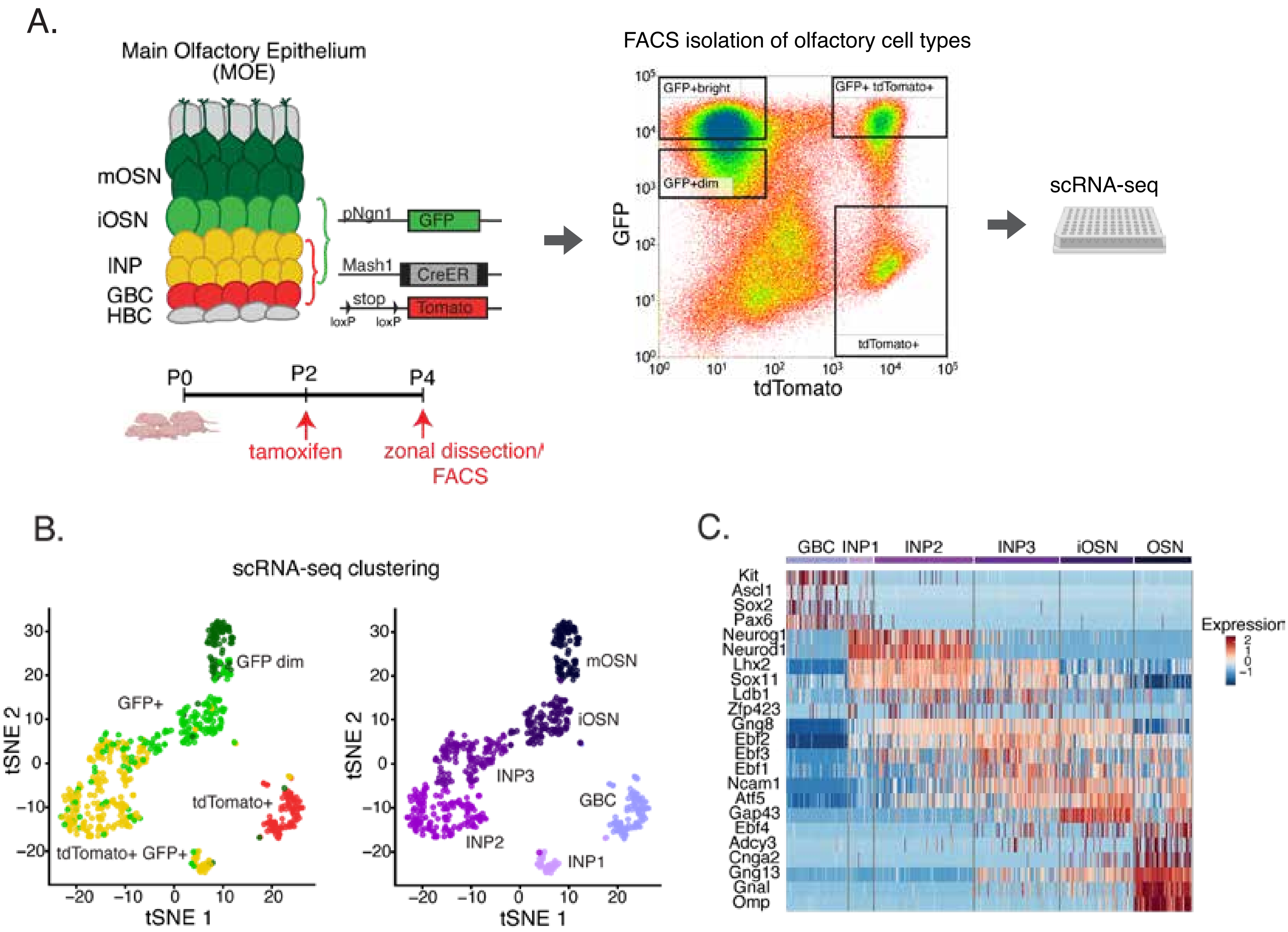

bioRxiv preprint doi: https://doi.org/10.1101/2023.03.15.532726; this version posted March 15, 2023. The copyright holder for this preprint (which was not certified by peer review) is the author/funder, who has granted bioRxiv a license to display the preprint in perpetuity. It is made available under aCC-BY 4.0 International license.

Supplemental Figure S2

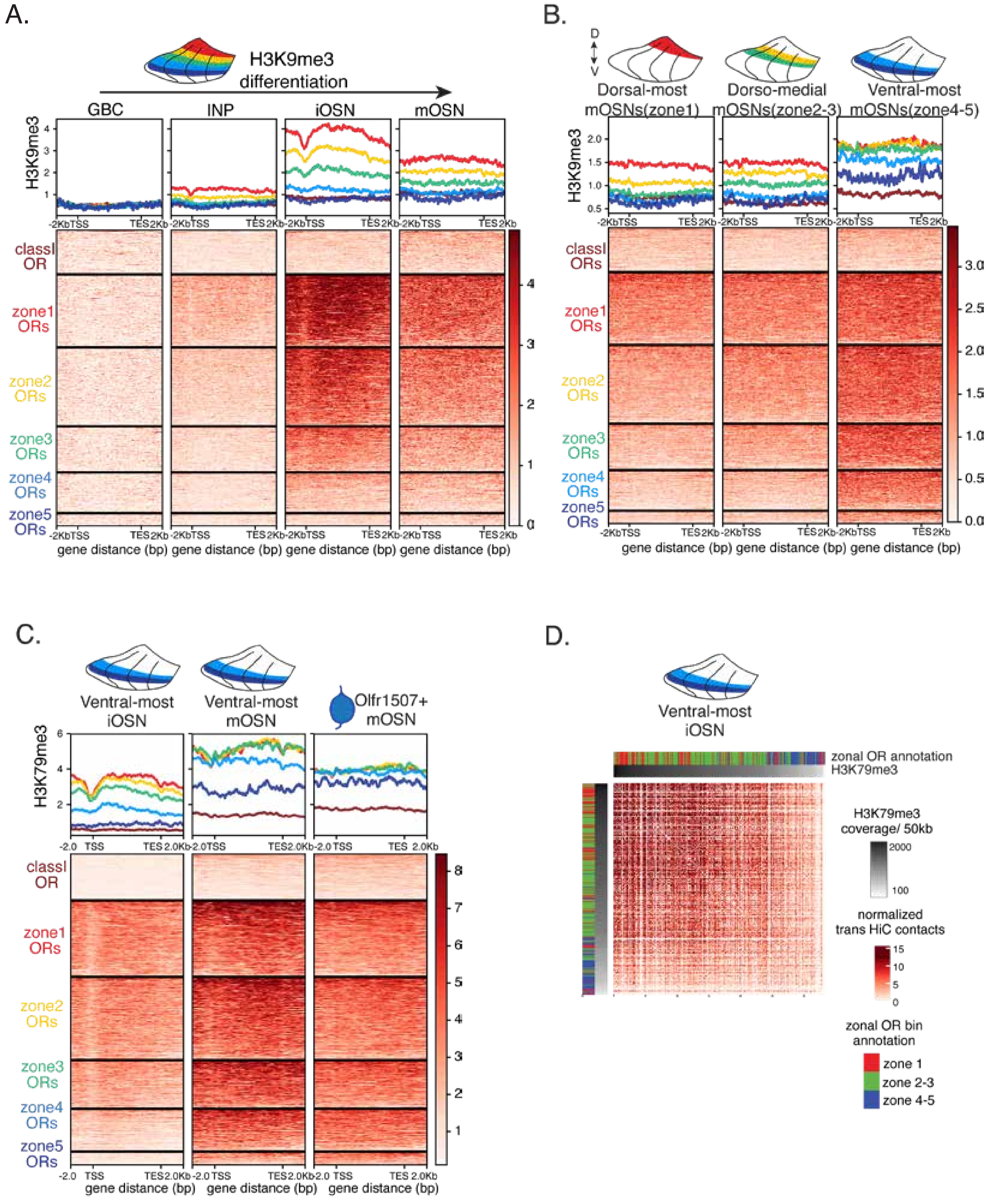

bioRxiv preprint doi: https://doi.org/10.1101/2023.03.15.532726; this version posted March 15, 2023. The copyright holder for this preprint (which was not certified by peer review) is the author/funder, who has granted bioRxiv a license to display the preprint in perpetuity. It is made available under aCC-BY 4.0 International license.

Supplemental Figure S3

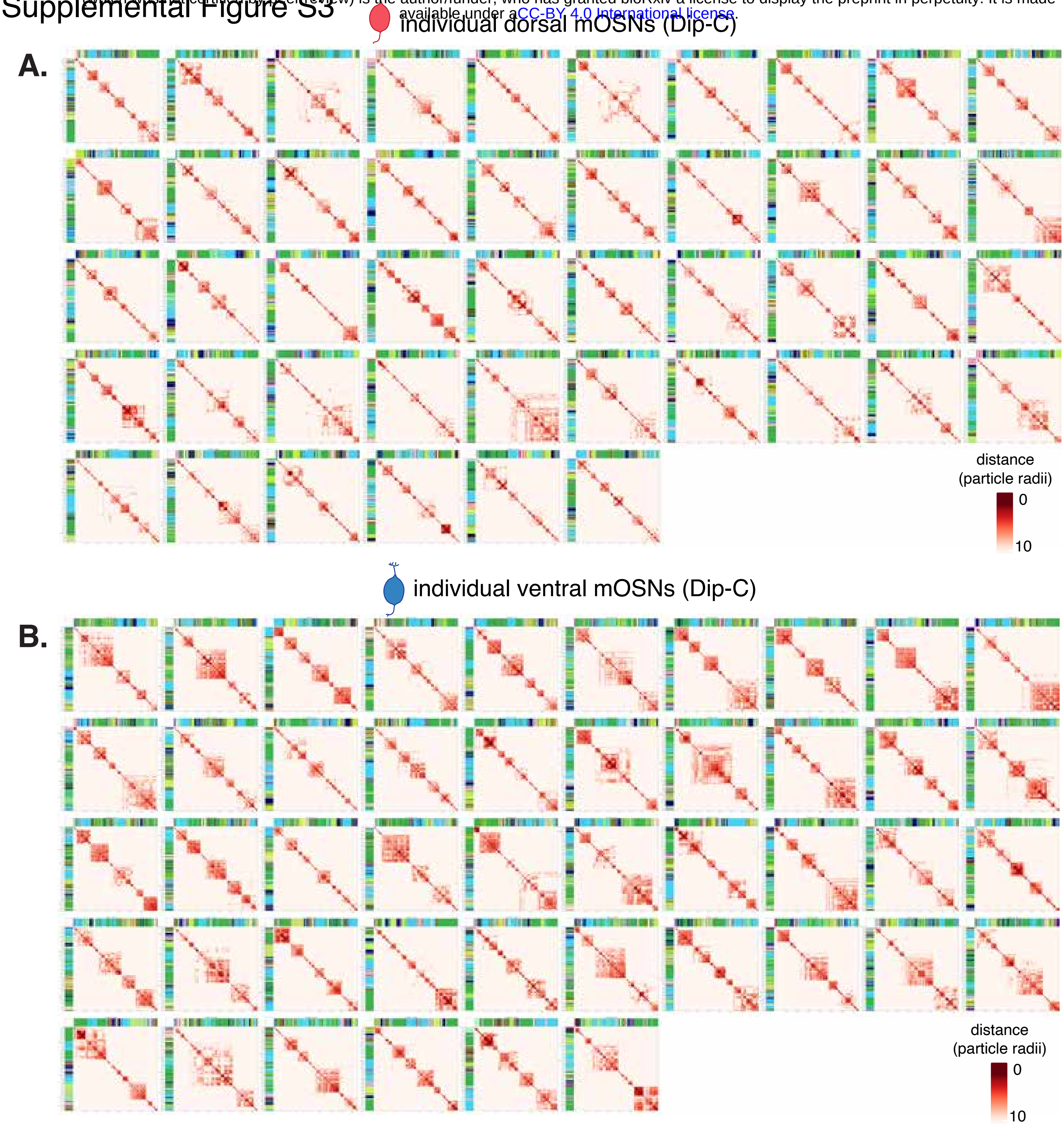

Supplemental Figure S4

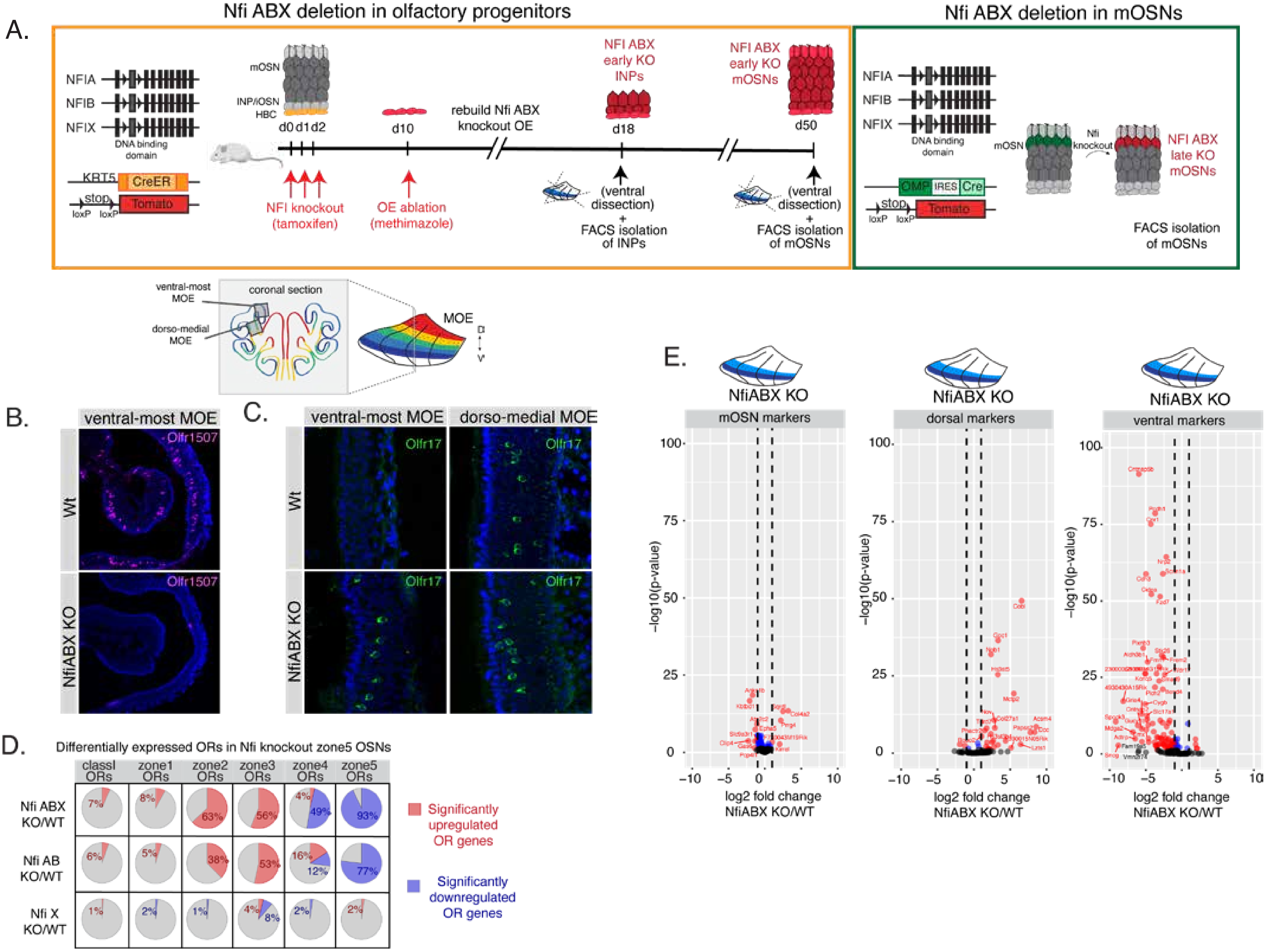

bioRxiv preprint doi: https://doi.org/10.1101/2023.03.15.532726; this version posted March 15, 2023. The copyright holder for this preprint (which was not certified by peer review) is the author/funder, who has granted bioRxiv a license to display the preprint in perpetuity. It is made available under aCC-BY 4.0 International license.

Supplemental Figure S5

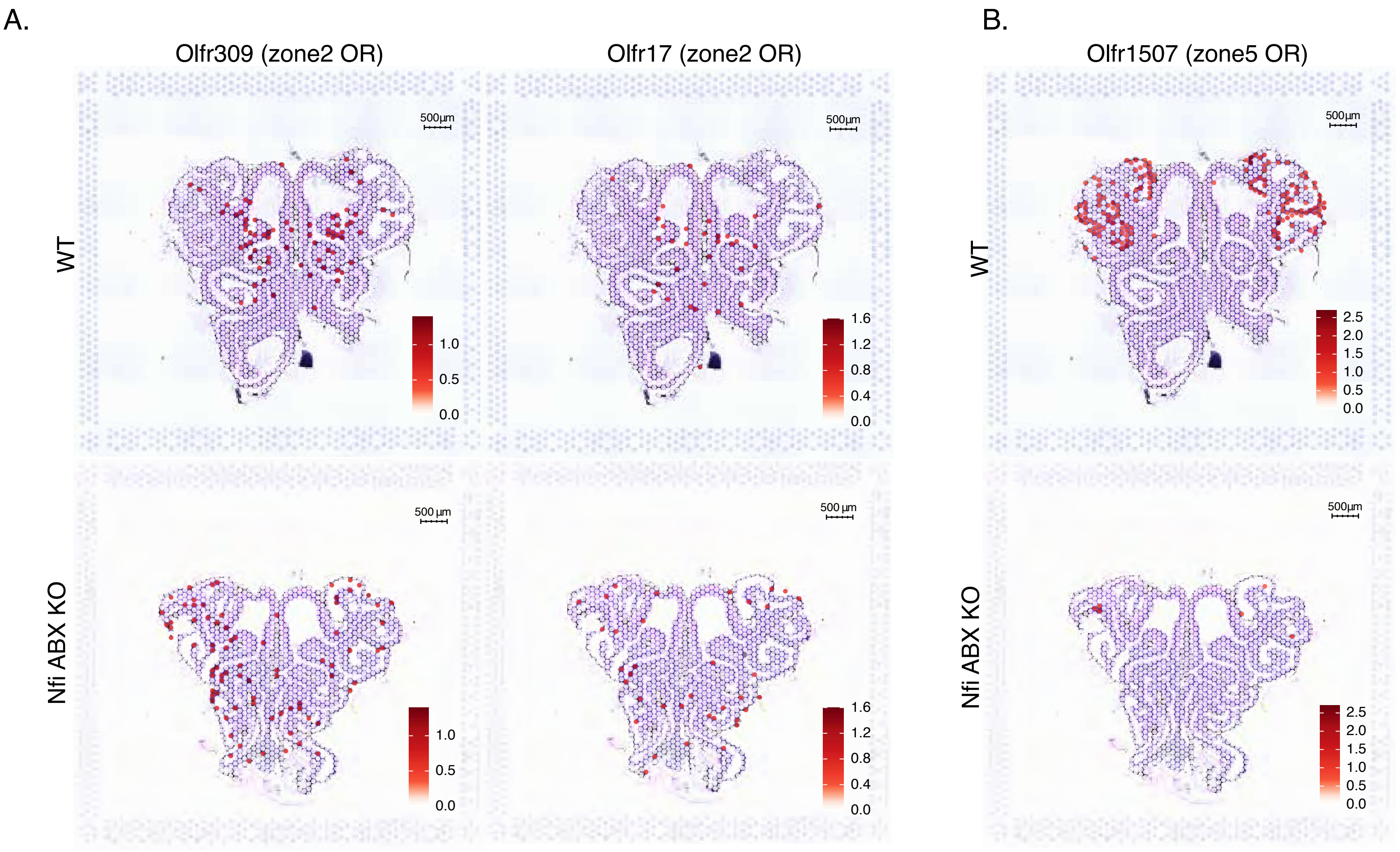

bioRxiv preprint doi: https://doi.org/10.1101/2023.03.15.532726; this version posted March 15, 2023. The copyright holder for this preprint (which was not certified by peer review) is the author/funder, who has granted bioRxiv a license to display the preprint in perpetuity. It is made available under aCC-BY 4.0 International license.

Supplemental Figure S6

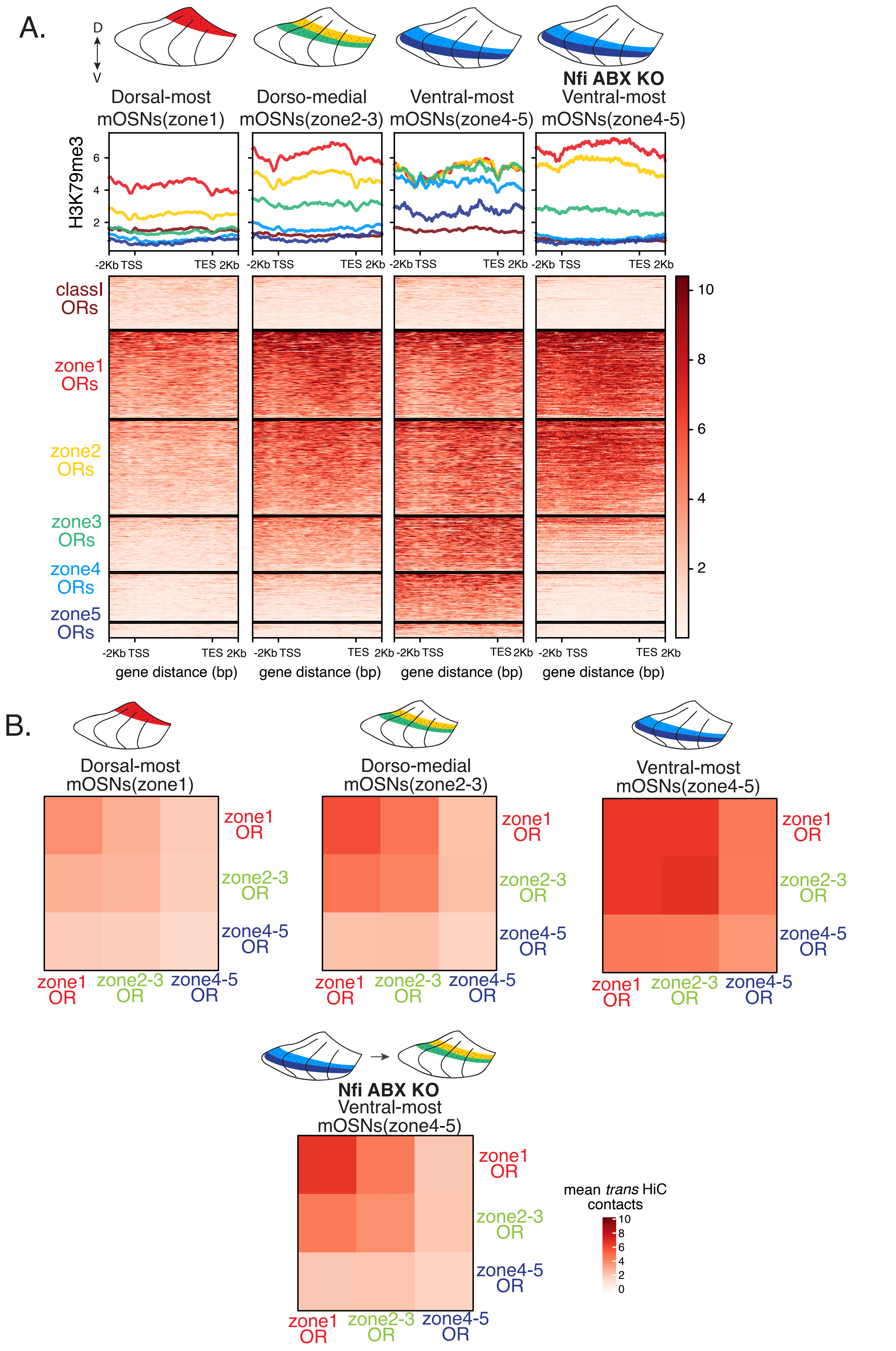

Supplemental Figure S7

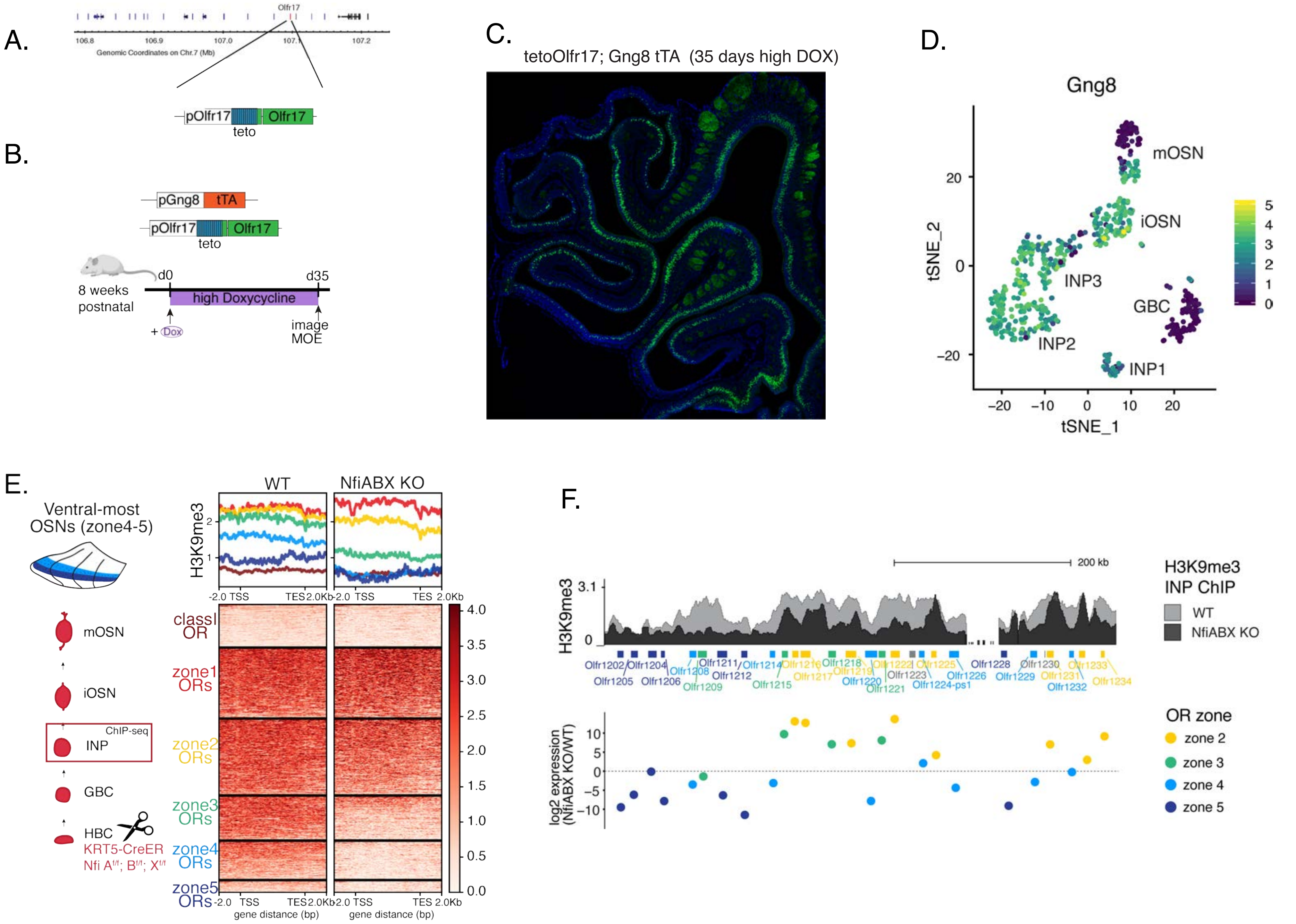

Supplement: Supplementary file 1 [file NIHPP2023.03.15.532726v1-supplement-1.pdf]
